# Hormonal Imbalances in Prader–Willi and Schaaf–Yang Syndromes Imply the Evolution of Specific Regulation of Hypothalamic Neuroendocrine Function in Mammals

**DOI:** 10.3390/ijms241713109

**Published:** 2023-08-23

**Authors:** Maria Camila Hoyos Sanchez, Tara Bayat, Rebecca R. Florke Gee, Klementina Fon Tacer

**Affiliations:** 1 School of Veterinary Medicine, Texas Tech University, 7671 Evans Dr., Amarillo, TX 79106, USA; 2 Texas Center for Comparative Cancer Research (TC3R), Amarillo, TX 79106, USA

**Keywords:** PWS, SYS, imprinting, hormone secretion, secretory granule, hypothalamus, neuroendocrine function, retromer, MAGEL2, NDN, SNORD116, MKRN3

## Abstract

The hypothalamus regulates fundamental aspects of physiological homeostasis and behavior, including stress response, reproduction, growth, sleep, and feeding, several of which are affected in patients with Prader–Willi (PWS) and Schaaf–Yang syndrome (SYS). PWS is caused by paternal deletion, maternal uniparental disomy, or imprinting defects that lead to loss of expression of a maternally imprinted region of chromosome 15 encompassing non-coding RNAs and five protein-coding genes; SYS patients have a mutation in one of them, *MAGEL2*. Throughout life, PWS and SYS patients suffer from musculoskeletal deficiencies, intellectual disabilities, and hormonal abnormalities, which lead to compulsive behaviors like hyperphagia and temper outbursts. Management of PWS and SYS is mostly symptomatic and cures for these debilitating disorders do not exist, highlighting a clear, unmet medical need. Research over several decades into the molecular and cellular roles of PWS genes has uncovered that several impinge on the neuroendocrine system. In this review, we will discuss the expression and molecular functions of PWS genes, connecting them with hormonal imbalances in patients and animal models. Besides the observed hormonal imbalances, we will describe the recent findings about how the loss of individual genes, particularly *MAGEL2*, affects the molecular mechanisms of hormone secretion. These results suggest that *MAGEL2* evolved as a mammalian-specific regulator of hypothalamic neuroendocrine function.

## 1. Introduction

**Figure 1:**
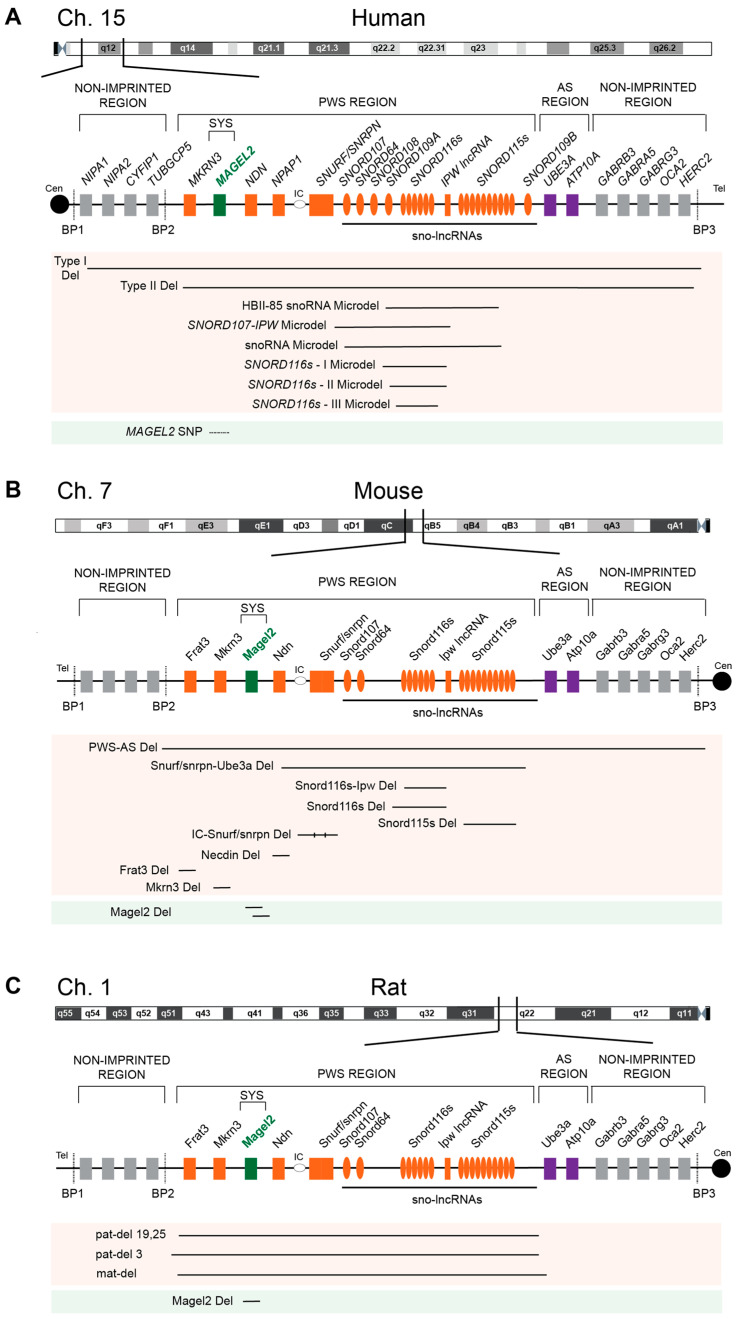
Gene map of PWS loci in humans (**A**), mouse (**B**), and rat (**C**) models with deletions reported in each species. Orange and green color—paternally expressed genes; purple color—maternally expressed genes; BP—break point; IC—imprinting center. The figure was generated based on [10,11,12].

Prader–Willi syndrome (PWS, OMIM #176270) and Schaaf–Yang syndrome (SYS, OMIM #615547) are rare autosomal-dominant, imprinted genetic disorders caused by the loss of one or more normally active paternal genes in the chromosomal region of 15q11-q13, called the PWS region. The two main molecular causes of PWS include paternal 15q11-q13 deletion, which is present in 65–75% of individuals with PWS, and maternal uniparental disomy in which both chromosome 15s are from the mother, present in 20–30% of cases (Figure 1A). The remaining individuals possess defects in the genomic imprinting center (IC) or chromosome 15 translocations or inversions [1,2,3]. PWS affects approximately one in 15,000–20,000 individuals with around 400,000 cases worldwide [4], while the prevalence of SYS is <1/1,000,000 [5]. Major clinical features of PWS include intellectual and physical disabilities, obesity, maladaptive behaviors, and several endocrine dysfunctions, like growth retardation and hypogonadism [6]. SYS shares several symptoms with PWS yet is distinct. The underlying genetic cause of SYS is the disrupted expression of *MAGEL2*, one of the protein-coding genes within the PWS region on chromosome 15, due to mutations in the paternal copy (Figure 1) [7,8,9].

**Figure 2:**
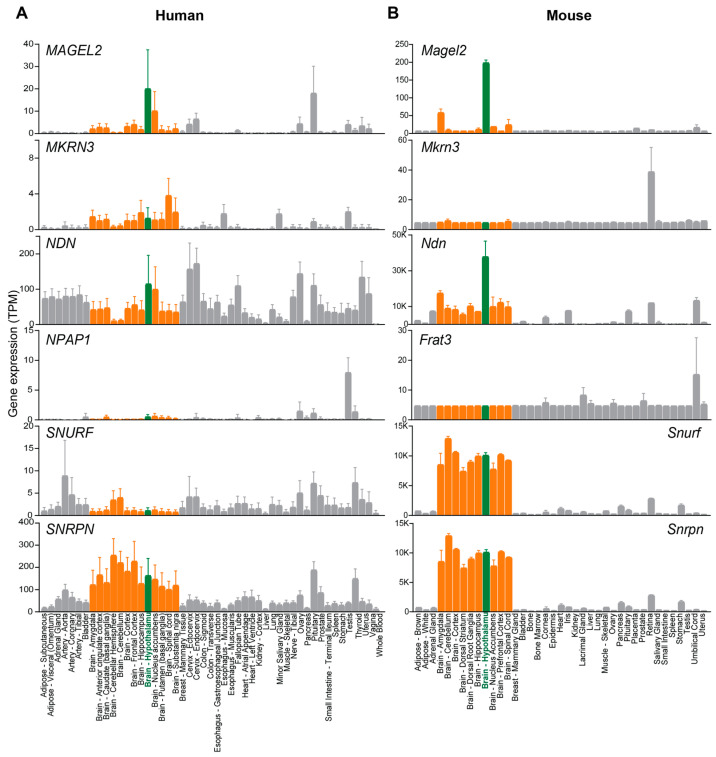
Gene expression of PWS region genes in humans and mice. (**A**) Human gene expression dataset extracted from GTEx Data Portal on 15 August 2021. (**B**) Dataset of gene expression in mice extracted from BioGPS on 15 August 2021 [18,19]. Orange and green color depict brain regions and hypothalamus, respectively.

The next section will provide an overview of the genes disrupted in PWS and SYS, including their expression and functions. We will also discuss individual mutations or deletions of some protein-coding and non-coding RNA genes that lead to hormonal imbalance, including precocious puberty in patients with *MKRN3* mutations and prohormone processing defects in *SNORD116* mutant mice [13,14]. Section three will describe the spectrum of clinical phenotypes for each disorder and phenotypes observed in animal models, several of which impinge on the neuroendocrine function of the hypothalamus. The fourth and final section will summarize the recent findings about the role of MAGEL2 in the production of neuropeptides and hormones in the regulation of hypothalamic hormone secretion in PWS. Given the emergence of the *MAGEL2* gene in eutherian mammals, its unique expression pattern (Figure 2), and recent insights into the physiological function of other *MAGE* genes in stress adaptation [15,16,17], we hypothesize that *MAGEL2* evolved to finetune hypothalamic regulation of physiological homeostasis and behavior and better adapt to environmental cues.

## 2. PWS-Associated Genes, Their Imprinting, and Expression Pattern

The critical region of PWS lies within a 6 Mb genomic locus on the long arm of chromosome 15 (Figure 1A). The maternally imprinted and paternally expressed genes within this PWS region encompass five protein-coding genes (*MKRN3*, *MAGEL2*, *NDN*, *NPAP1*, and *SNURF-SNRPN*) and a family of six non-coding, small nucleolar RNA (snoRNA) genes. Unlike the single copy snoRNA genes, *SNORD116/HBII-85* and *SNORD115/HBII-52* are snoRNA gene clusters with multiple copies, 29 and 48 copies, respectively, though the exact number may vary among individuals (Figure 1A) [20,21,22]. Though paternal deletion of a single gene or even three (i.e., *MAGEL2*, *MKRN3*, and *NDN*) does not result in PWS [23], research into the individual genes has provided insight into the role of each gene in the complex symptomology of PWS.

The syntenic PWS regions on chromosome 7C in mice and chromosome 1 in rats are generally organized and imprinted similarly to the human PWS region (Figure 1B,C) [3,12,24,25]. The positional conservation and gene organization, including the imprinting pattern between mice, rats, and humans, imply evolutionarily conserved physiological functions of this locus in mammals. Therefore, researchers have taken considerable efforts to recreate the pathogenesis of PWS in mouse and rat models [11]. Murine models of PWS unveiled the contribution of each affected gene in this multi-faceted disease and enabled the establishment of the minimal critical genomic region responsible for core symptoms, highlighting the importance of non-protein coding genes in the PWS locus [24]. Although the underlying disease-causing mechanisms of PWS remain widely unresolved and existing models do not fully capture the entire spectrum of the human PWS disorder, continuous improvements of genetically engineered mouse and rat models have proven to be very powerful and valuable tools in PWS research [26].

### 2.1. MKRN3

*MKRN3*, a highly conserved PWS-associated gene sharing 82% similarity between humans and mice, is ubiquitously expressed in human and mouse tissues with high levels in the brain and testis (Figure 2) [21,27,28]. The hypothalamic *MKRN3* expression is high early in life and decreases before puberty initiation, an evolutionarily conserved pattern observed in mice, rats, and non-human primates [29]. MKRN3 belongs to the Makorin family of proteins that contain two to four C3H zinc finger domains, a unique Cys-His configuration, and a RING zinc finger domain that is critical for the activity of the RING subfamily of E3 ubiquitin ligases [28,30]. Paternal deletion or loss-of-function mutations in *MKRN3* are thought to contribute to hypogonadism, infertility, and the rare cases of central precocious puberty (CPP) in PWS patients [13,21,31,32,33]. CPP is characterized by elevated expression and secretion of hypothalamic gonadotropin-releasing hormone (GnRH), resulting in the early development of secondary sexual characteristics [13,34]. *Mkrn3* knockout mice phenocopy many symptomatic features of human CPP [35]. Although MKRN3’s function in regulating puberty initiation in mammals is not completely understood, CPP-associated mutations in *MKRN3* result from reduced expression or loss of E3 ubiquitin ligase function. MKRN3 ubiquitinates MBD3 (methyl-DNA binding protein 3) and epigenetically silences *GNRH1* [35]. Furthermore, MKRN3-mediated ubiquitination of poly(A)-binding proteins destabilizes *GNRH1* mRNA in the hypothalamus [34]; thus, MKRN3 regulates GnRH on the transcriptional and translational level. MKRN3 also inhibits puberty onset through interaction with other proteins, including IGF2BP1 and NPTX-1 [36,37,38]. Additionally, an in vitro luciferase assay showed that MKRN3 inhibited the expression of kisspeptin and neurokinin B, neuropeptides that stimulate GnRH secretion [29], though only neurokinin B protein levels were increased in *Mkrn3* knockout mice [36]. In short, MKRN3 is a neuroendocrine inhibitor upstream of GnRH, and *MKRN3* loss-of-function mutations are the main genetic cause of CPP, including in PWS patients [34,35,39,40].

### 2.2. MAGEL2

*MAGEL2* is a member of the melanoma-antigen (MAGE) gene family, which expanded from one gene in lower eukaryotes to more than 40 genes in eutherian mammals [17]. *MAGEL2* encodes for a regulator protein of an E3 ubiquitin ligase and affects the retromer-dependent endosomal protein recycling [7,41,42,43]. *MAGEL2* is highly expressed in the central nervous system, especially in the hypothalamus (Figure 2) and placenta [7,15]. *Magel2*-null mice recapitulate many PWS features, like poor sucking and obesity [44,45]. The loss of *MAGEL2* leads to decreased neuropeptide and hormone production and impaired hypothalamic secretion, which will be discussed later in further detail.

### 2.3. NECDIN

Necdin (encoded by *NDN*) is another member of the MAGE family within the PWS region. *NDN* is highly expressed in certain regions of the brain, such as the locus coeruleus and hypothalamus and placenta (Figure 2) [17,46,47,48]. In particular, *NDN* is highly expressed in GnRH neurons in the mature hypothalamus [49]. Necdin plays an integral role in neuronal differentiation [47], so Necdin deficiency leads to widespread nervous system abnormalities [50]. Deletion of *Ndn* in mice recapitulates several PWS symptoms, including neonatal mortality, altered pain threshold, hypogonadism, and sensory–motor defects [24,50,51], and significantly reduces the quantity of hypothalamic GnRH neurons [48,52]. Muscatelli et al. [53,54] reported a significant reduction in oxytocin-expressing neurons in the lateral parts of the paraventricular hypothalamic nucleus of *Ndn*-deficient mice. *Necdin*-deficient mice also exhibit disturbed migration of serotonin neuronal precursors and increased serotonin transporter activity that causes apnea, making *Ndn* knockout mice the only model that reproduces the respiratory challenges of the PWS [51,54,55]. Further, Necdin and Magel2 together were shown to control leptin receptor sorting and degradation through a ubiquitin-dependent pathway, including E3 ubiquitin ligase Rnf41, deubiquitinase Usp8, and protein Stam1, contributing to obesity in PWS [45]. More recently, Necdin was reported to regulate the stability of BMAL1, one of the core transcription regulators of the circadian rhythm, potentially contributing to the disturbed circadian rhythm observed in patients [46].

### 2.4. NPAP1

The imprinted *NPAP1* is a primate-specific gene encoding a nuclear pore complex-associated protein from a POM121-related family of retrogenes with testis-specific expression, a unique pattern among the PWS genes (Figure 2) [56,57,58]. Interestingly, *NPAP1* is the only PWS gene not conserved in rodents (Figure 1). Rather, the PWS syntenic region on chromosomes 7 and 1 in mice and rats, respectively, contains another coding gene, *Frat3*, that is potentially involved in WNT signaling during embryonic development (Figure 1B,C) [59].

### 2.5. SNURF/SNRPN

*SNURF/SNRPN* (SNRPN upstream reading frame (SNURF)/small nuclear ribonucleoprotein polypeptide N (SNRPN)) is a complex gene locus belonging to the SNRPN SmB/SmN family. The protein plays a role in pre-mRNA processing, tissue-specific alternative splicing events, and transcript production [4]. The *SNURF/SNRPN* gene is a bicistronic transcript that encodes two proteins and also contains the *snoRNA* genes (Figure 2) [11,25,60,61]. Chromosomal deletions that affect the *SNRPN* upstream exons and the imprinting center (IC) cause PWS by impairing the allele-specific expression of genes normally subject to the imprinting control [62]. It has also been reported that even a single small deletion or single-nucleotide variant involving *SNURF/SNRPN* causes major symptoms of PWS including hypotonia, dysmorphic features, intellectual disability, and obesity [63,64].

### 2.6. SNORD116

The non-coding RNA molecule *SNORD116* is highly expressed in the brain [65], and clinical evidence from rare patients with *SNORD116* deletions or translocations indicates that the *SNORD116* cluster is crucial for most of the PWS phenotypes [21,66,67,68,69,70,71]. In mice, global or selective deletion of *Snord116* from hypothalamic neurons causes low birth weight, increased weight gain in early adulthood, increased energy expenditure, and hyperphagia [72]. One group reported that *Snord116* paternal knockout (*Snord116^m+/p−^*) mice also had reduced transcript levels of the prohormone convertase PC1 (encoded by *Pcsk1*), impairing prohormone processing and possibly causing the major neuroendocrine features of PWS [14]. Chen et al. [73] also observed a reduction in PC1 protein levels in pancreatic islets from *Snord116^m+/p−^* mice, although a follow-up study found no differences in the hypothalamic *Pcsk1* transcript levels in *Snord116^m+/p−^* mice [74].

Besides PWS and SYS, Angelman syndrome (AS, OMIM#105830) is another imprinting disorder that is caused by genetic variation in the same region of chromosome 15 (Figure 1). Ataxia, happy demeanor, and sleeplessness are some of the symptoms observed in individuals with AS [75,76]. In AS, the maternal copy of the genes in 15q11-q13 is missing, while the paternal copy is inactivated in PWS and SYS [7,76]. The PWS region is maternally imprinted, and genes must be expressed from the paternal chromosome. In contrast, the adjacent AS region is paternally imprinted, and encoded genes must be expressed from the maternal chromosome.

### 2.7. Genomic Imprinting

Genomic imprinting in PWS and AS causes a monoallelic expression of genes and is regulated by a bipartite IC, composed of the PWS-IC and AS-IC, that establishes local imprinting regulation of multiple genes within the 15q11-q13 region (Figure 1) [77,78]. The PWS-IC comprises a CpG island and is associated with the 5′ flanking region, the first exon and 5′ end of the first intron of *SNRPN* [79]. The AS-IC is located 35 kb upstream of the *SNRPN* promoter [80]. The PWS-IC is differentially methylated on the maternal allele, with the paternal allele remaining in an open, unmethylated state [60,78,81,82,83]. Interestingly, deletion of AS-IC on the maternal allele also leads to the biallelic unmethylation of neighboring PWS-IC, suggesting that AS-IC contributes to establishing the methylation state and closed chromatin structure of PWS-IC [78,84]. Paternally inherited deletion of the PWS-IC results in loss of expression of *MAGEL2* and other PWS genes [3,79]. For example, upon deletion of murine paternal PWS-IC, Brant et al. [85] noted a two-fold increase in the methylation level of CpG sites at differentially methylated regions (DMRs) of paternally expressed PWS genes, including *Magel2*, while deletion of maternal PWS-IC resulted in no methylation changes.

Although there are contradictory findings regarding when the maternal PWS-IC becomes methylated, in oocytes or post-fertilization, most results suggest that methylation occurs after the blastula stage [86,87,88,89]. A proposed model for PWS imprinting regulation suggests that methyl groups are removed from PWS-IC during both spermatogenesis and oogenesis, and then AS-IC interacts with PWS-IC to facilitate de novo methylation of the maternal PWS-IC after fertilization [79]. After fertilization and establishment of methylation of the maternal PWS-IC, the unmethylated paternal PWS-IC functions as a promoter for the *SNRPN* transcription unit and acts at long distances to activate transcription of *MAGEL2*, amongst others [79]. However, the exact mechanism and proteins involved in this intricate process are unclear.

### 2.8. Expression Pattern

In addition to the epigenetic regulation of imprinted genes in the PWS region (Figure 1), the distinct tissue expression patterns of these genes (Figure 2) clearly suggest specific transcriptional regulation; however, the details of potential transcription factors involved are almost completely unknown. Interestingly, *MAGEL2* and *NDN* are expressed at the highest levels in the brain (especially *MAGEL2* in the hypothalamus), pituitary, and placenta (Figure 2) [15,90], tissues linked to the evolution of genomic imprinting in eutherian mammals. For instance, most of the imprinted genes (~230 genes known so far) are expressed in the placenta and several now have well-known roles in placental biology, fetal growth, and homeostasis of pregnancy [91,92]. The functional convergence of many imprinted genes on the placenta is in line with the hypothesis that genomic imprinting evolved in mammals because of the conflicting interests of maternal and paternal genes in relation to the transfer of nutrients from the mother to her offspring [93,94]. Very little is known about the role of the PWS genes in the placenta and warrants future investigation.

**Figure 3:**
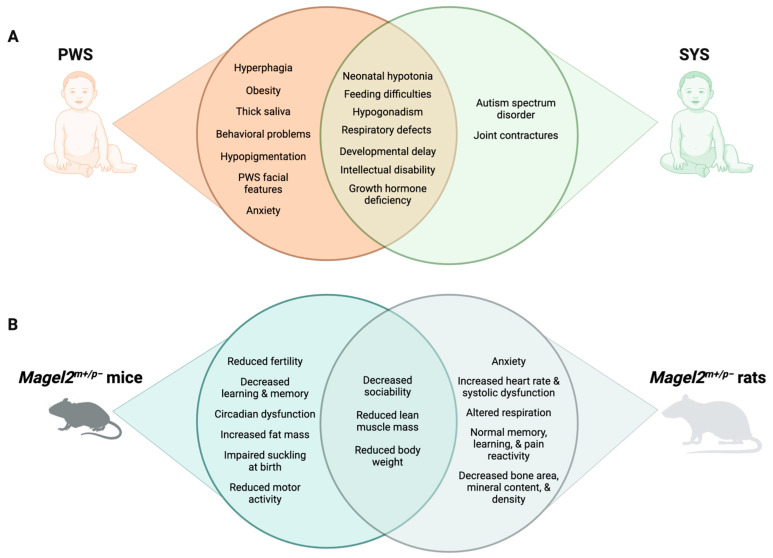
Comparison of PWS and SYS phenotypes in humans (**A**) and rodent models (**B**).

Furthermore, genomic imprinting is increasingly appreciated for its role in the nervous system. Outside of the placenta, the brain is one of the adult tissues with the largest number of expressed imprinted genes [95,96]. In a recent tissue expression analysis on a single-cell level, imprinted genes were found over-represented in murine hypothalamic neurons [97]. These data support the previously suggested role of imprinted genes in the neuroendocrine hypothalamic regulation of diverse physiological functions [98]. Indeed, the first insights into the physiology of imprinted gene function were derived from studying neuroendocrine symptoms in PWS patients and animal models [99]; however, we are only beginning to understand the underlying molecular mechanisms [14,73]. Through connections with the pituitary, the hypothalamus regulates hormone release in distal endocrine glands, including adrenals, thyroid, and gonads, to control key physiological processes, like stress response, growth, and reproduction. In addition, the hypothalamus makes neural connections via the autonomic nervous system and other pathways to regulate sleep, body temperature, and feeding [100], several of which are disturbed in PWS and SYS (Figure 3).

To summarize, mouse models and unique genetic backgrounds of patients revealed how single or multiple genes contribute to the development of PWS and SYS symptoms. The unique expression pattern of PWS genes (e.g., *MAGEL2*), their evolutionary appearance, and their molecular and cellular roles (discussed in more detail in the next sections) suggest that some of the PWS genes evolved in mammals as tissue-specific regulators of hypothalamic neuroendocrine function.

## 3. Hormonal Imbalance in PWS and SYS on an Organismal Level

### 3.1. Symptoms and Treatment of PWS

Though the complex symptomology of PWS affects multiple systems (i.e., neurological, cognitive, and endocrine), its developmental trajectory was historically divided into two phenotypically opposing stages: hypotonia and failure to thrive in infancy (Stage 1), followed by hyperphagia that leads to obesity in childhood (Stage 2) [101]. Both stages suggest dysfunction of hypothalamic feeding regulation, but the mechanistic details behind the switch remain enigmatic. More recently, a longitudinal study of patients identified seven nutritional phases, highlighting the gradual, complex progression of PWS [102]. In the beginning, PWS is characterized by growth restriction and decreased fetal activity in utero (Phase 0), followed by severe neonatal hypotonia with feeding difficulties like poor sucking and swallowing problems (Phase 1a) [102]. From about 9–25 months of age, infants exhibit a normal appetite and grow appropriately along their growth curve (Phase 1b) [102]. Around two years of age, PWS patients start gaining weight without an increase in appetite (Phase 2a) but begin to show an increased interest in food a year or two later (Phase 2b) [102]. From the age of about eight years into adulthood, the dominating feature of PWS is hyperphagia, accompanied by food-seeking behaviors and a general lack of satiety (Phase 3). If not externally controlled by calorie management and restricted food access, rapid and excessive weight gain, starting as early as 1 year of age, will lead to obesity and all its associated comorbidity risks [101,103]. Up to 25% of adults with PWS have type 2 diabetes with an average age of onset of 20 years [6,104], which is likely related to morbid obesity and consequent insulin resistance [105]. Some adults may move beyond Phase 3 if they no longer have an insatiable appetite and can feel full (Phase 4) [102].

Beyond food-related symptoms, developmental delays and diminished growth with small hands and feet become apparent during the early phases of PWS (Figure 3A) [106]. Dysautonomia, hormonal deficiencies, intellectual delay, and difficulties with learning and social communication also emerge during childhood [103,107,108]. Short stature, if not evident in childhood, is almost always present during the second decade in the absence of growth hormone replacement [6]. Furthermore, PWS patients often exhibit behavioral problems, including compulsive tendencies, temper tantrums, outbursts, and severe skin picking [103,109]. Sleep abnormalities and scoliosis are common in PWS [106]. In addition, psychiatric illnesses, including depression, anxiety and mood disorders, psychosis, and autism spectrum disorder (ASD), are associated with PWS [110,111,112,113].

PWS patients have a shorter life expectancy with an average age of death around 29 years [114]. The overall mortality rate is 2.7%, highest among PWS patients aged 0–2 years and lowest among those aged 9–17 years [115]. In children, the most common causes of death are respiratory illnesses due to a narrow upper airway, central hypotonia, hypoventilation, and febrile illnesses [1]. In adults, the most common causes of death are complications from hyperphagia and obesity-related comorbidities, such as cardiac disease/failure, pulmonary thromboembolism, gastrointestinal problems, and diabetes [104,116].

There is no cure for PWS, so weight control is one of the main goals in the treatment [117]. Management of PWS is symptomatic and supportive, emphasizing food intake control, hormone replacement therapies, special education, and behavior management [118]. Growth hormone replacement therapy, which is the only U.S. Food and Drug Administration (FDA)-approved treatment for PWS, can improve body composition, physical strength, and cognitive level [119]. Oxytocin, oxytocin analogs, and molecules targeting the ghrelin system are currently under investigation as potential treatments for PWS [120]. Medications like topiramate, metformin, and naltrexone–bupropion have yielded some favorable results (e.g., weight reduction and improved behavioral conditions) in PWS patients [117]. Non-pharmacological strategies to manage hyperphagia and obesity include rigid control of diet, restricted access to food, and regular exercise plans [103].

### 3.2. Symptoms and Treatment of SYS

Truncating point mutations of the paternal allele of *MAGEL2* cause SYS [121,122,123]. Though PWS and SYS share several phenotypes, SYS becomes more clinically distinct throughout childhood and adolescence [123]. Some of the overlapping phenotypes include neonatal hypotonia, feeding difficulties, hypogonadism, short stature, developmental delay, and intellectual disability (Figure 3A) [7,122]. One of the unique features of SYS is the presence of joint contractures, which range in severity from mild contractures of the distal phalanges to lethal arthrogryposis [17,123,124,125]. There is also a higher prevalence of ASD in SYS patients (75–85% compared to about 25% in PWS patients) [8,113,121,122,123,126]. Furthermore, the profound hyperphagia and morbid obesity that are hallmark features of PWS are not usually associated with SYS [122]; however, weight gain and food-seeking behavior are present in adult SYS patients [5]. Hypogonadism may be less frequent in SYS, as it is only reported in 15–25% of females and 55–65% of males with SYS compared to 70–100% of PWS patients [49,122]. Other PWS features that are not commonly observed in SYS patients include hypopigmentation, PWS characteristic facial features, small hands and feet, thick saliva, and behavioral problems [21].

Intriguingly, patients with deletions encompassing the whole *MAGEL2* gene, but not the *SNORD116* cluster, were reported to have much milder phenotypes than those with point mutations in *MAGEL2* that cause SYS [23,127]. The lack of joint contractures, autism characteristics, and hyperphagia suggest that complete deletion of the paternal copy of the *MAGEL2* gene and promoter could lead to leaky expression of the maternal copy of *MAGEL2*, as observed in mice [128]. In contrast, SYS-associated truncating mutations in the single-exon *MAGEL2* gene may not cause nonsense-mediated mRNA decay but produce a truncated MAGEL2 protein that could have neomorphic effects [121].

As with PWS, SYS has no cure and treatment focuses on managing symptoms to improve quality of life. The overall life expectancy of people with SYS is shortened due to an increased risk of fatal complications, primarily during infancy and childhood. Many infants experience respiratory distress due to central or obstructive apnea, requiring invasive or non-invasive assisted ventilation. Poor weight gain and persistent feeding issues may be addressed with feeding therapy or supplemental tube feeding [122]. Treatment plans for SYS may also include growth hormone replacement therapy for short stature and standard therapies for gastroesophageal reflux, constipation, and skeletal abnormalities [122,129,130].

## 4. Hormonal Imbalance in PWS and SYS on a Molecular Level

PWS and SYS are characterized by having a marked hormonal imbalance that generates different phenotypes. Since the hypothalamus receives and orchestrates a variety of signals to coordinate whole-body homeostasis through the hypothalamus–pituitary–target endocrine gland axis, impaired hypothalamic development and function appear to be present in many of the PWS and SYS clinical phenotypes. This hypothalamic dysfunction manifests through the various endocrine abnormalities observed in patients, including growth hormone deficiency, hypogonadism, premature adrenarche, hypothyroidism, central adrenal insufficiency, low bone mineral density, and impaired glucose tolerance (Figure 3 and Table 1) [1,106,131,132,133].

### 4.1. Growth Hormone

Growth hormone (GH) deficiency is present in the majority of PWS cases (up to 74%) and is assessed through measuring the level of insulin-like growth factor 1 (IGF-1), the major mediator of GH [103,119,180]. Short stature, small hands and feet, low motor strength, increased fat mass, and decreased movement and energy expenditure are symptoms associated with growth hormone deficiency [21]. SYS patients tend to exhibit a combination of short stature, high-fat mass, and low IGF-1 levels, suggesting a growth hormone deficiency like PWS [123,131]. *Magel2* depletion in mice also leads to a disrupted hypothalamus–pituitary axis regulating growth hormone, including a decrease in somatostatin in the hypothalamus and depletion of growth hormone in the pituitary (Table 1) [73]. Mechanistically, recent data suggest that MAGEL2 regulates the core machinery of the regulated secretory pathway in the hypothalamus by directing the E3 ubiquitin ligase activity of tripartite motif-containing 27 (TRIM27) and, thus, controlling multiple endocrine functions [42,43,73]. The current understanding of the MAGEL2 function will be further discussed in the last section. *Snord116* also impacts the secretion of several hormones and neuropeptides in the hypothalamus, pancreas, and stomach by regulating the expression of PC1, which is involved in the maturation of prohormones, including GH releasing hormone (GHRH), insulin, and ghrelin (Table 1) [14].

### 4.2. Hypogonadism and GnRH

Hypogonadism is present in both sexes and both syndromes. It manifests as genital hypoplasia, incomplete pubertal development, and infertility in the majority of cases [181]. Hypogonadism is often associated with low serum gonadotropins, likely due to hypothalamic dysfunction [6,131]. The molecular functions of several PWS genes impinge on the regulation of GnRH and the GnRH–gonadotropin–sex hormones endocrine axis (Table 1). *NDN* is highly expressed in GnRH neurons [49], and its deletion in mice significantly reduces the quantity of hypothalamic GnRH neurons and recapitulates PWS-associated hypogonadism [24,48,50,51,52]. Necdin is also proposed to activate GnRH transcription by preventing MSX-mediated repression of GnRH [48]. *Magel2* deletion in mice leads to impaired fertility [7,182] and reduced levels of hypothalamic GnRH and pituitary luteinizing hormone (LH) (Table 1) [73]. Through its E3 ubiquitin ligase activity, MKRN3 serves as a negative regulator of GnRH secretion on the transcriptional and translational level, and *MKRN3* depletion contributes to hypogonadism, infertility, and central precocious puberty (CPP) in PWS [13,21,32,33,34]. Interestingly, ubiquitination emerges as a unifying molecular mechanism underlying the finetuning function of several PWS-associated genes, including *NDN, MAGEL2,* and *MKRN3* [34,37,43,45,183].

### 4.3. Hypothyroidism

Central hypothyroidism, with a normal thyroid-stimulating hormone value and a low free thyroxine level, has been documented in up to 25% of people with PWS, with a mean age of diagnosis and treatment of two years [6,184]. The thyrotropin-releasing hormone (TRH) neurons in the paraventricular nucleus (PVN) of the hypothalamus that are part of the hypothalamic–pituitary–thyroid axis play a critical role in mediating changes in metabolism and thermogenesis [185]. Accordingly, the sensations of cold or excessive sweating in PWS patients are indicative of hypothalamic dysfunction [131], which is supported by the decreased hypothalamic TRH levels [73] and circulating T4 levels [186] reported in *Magel2* depleted mice (Table 1). These results suggest the importance of MAGEL2 under different physiological conditions for regulating whole body response to diverse stressors, including cold and changes in nutritional status [17].

### 4.4. Adrenal Insufficiency

Central adrenal insufficiency, which is caused by insufficient production of adrenocorticotropic hormone (ACTH) by the pituitary, occurs in about 5% of PWS patients [21,150]. The introduction of growth hormone therapy can precipitate an adrenal crisis in individuals with incipient adrenal insufficiency by accelerating the peripheral metabolism of cortisol, which may explain the correlation between the incidence of sudden death at the beginning of growth hormone treatment and adrenal insufficiency in individuals with PWS [187]. Although an early study indicated a high prevalence of adrenal insufficiency [148], subsequent studies with larger patient samples reported normal cortisol responses to ACTH stimulation tests, indicating that adrenal insufficiency in PWS is rare [149,188,189]. Female *Magel2*-null mice failed to respond to hypoglycemia with increased corticosterone, suggesting MAGEL2 deficiency might contribute to adrenal insufficiency [186].

### 4.5. Ghrelin

The ghrelin and oxytocin systems are impaired in most patients [120]. Ghrelin is a peripheral hormone produced in the stomach and is the endogenous ligand of the growth hormone secretagogue receptor in the hypothalamic arcuate nucleus that regulates food intake and satiety [106,190]. PWS patients and mouse models, such as *Snord116del^m+/p−^* mice, exhibit elevated ghrelin levels that likely contribute to obesity and hyperphagia (Table 1) [14,164,165]. However, higher ghrelin levels reported in individuals in the early phases of PWS (i.e., before the onset of hyperphagia) suggest that ghrelin is not fully responsible for the switch to the hyperphagic phase of PWS [106,191].

### 4.6. Oxytocin and Other Neuropeptides

The neuropeptides oxytocin (OXT) and arginine vasopressin (AVP) are evolutionarily highly conserved mediators in the regulation of complex social cognition and behavior. Both molecules are synthesized in overlapping regions of the hypothalamus, primarily in large magnocellular neurons of the supraoptic and paraventricular nuclei. These neurons project their axons to the posterior pituitary, where the peptides are stored in secretory granules until released into the peripheral circulation [192]. Oxytocin impacts several physiological functions (e.g., sexual response, uterine contractions, and lactation), as well as social behaviors like bonding [193]; thus, defects in oxytocin may contribute to several symptoms, such as poor suckling response at birth, hyperphagia with food addiction, poor social skills, and emotional dysregulation [120]. Multiple reports have demonstrated oxytocin abnormalities in PWS patients, including fewer oxytocin-producing neurons [194], altered oxytocin levels in plasma (i.e., lower in adults [194,195] but higher in children [196]), and increased oxytocin levels in cerebrospinal fluid [197,198,199]. Dysregulation of the oxytocin system may go beyond the altered expression of oxytocin, as Ates et al. [199] demonstrated that drastic changes in the synaptic excitation/inhibition balance led to suppression in overall activity in oxytocin-expressing neurons. Many of these phenotypes have been recapitulated in mouse models of PWS, implicating *Magel2* and *Snord116* in oxytocin secretion [14,44,73,140]. Oxytocin administration in *Magel2* mutant mice produced beneficial effects, including restoration of normal feeding behavior after birth [44], thermosensory response, maternal pup retrieval [200], and hippocampal alterations and social memory in adulthood [201]. Given the results from animal studies, oxytocin therapy is one of the important therapeutic strategies for PWS and SYS children [158].

Besides oxytocin, researchers have identified decreases in several other neuropeptides relevant to PWS (Table 1). Lower levels of AVP, which regulates the tonicity of body fluids and affects behaviors related to social interactions, have been reported in *Magel2*-null mice and PWS patients [65,73,202]. Orexin/hypocretin, which is important for sleep regulation, food intake, and energy balance, is also altered in PWS patients and mouse models (Table 1) [153,203]. Low levels of orexin in cerebrospinal fluid [153,155] and reduced levels of acetylcholinergic neurons in the peduncle-pontine tegmental nucleus of PWS patients [204] indicate that primary hypothalamic dysfunction may cause the apnea and sleep abnormalities observed in PWS patients.

Given that PWS represents one of the most common rare genetic disorders, the PWS and AS imprinted gene cluster on 15q11-q13 has attracted many researchers’ interest with the primary aim of helping patients. At the same time, the insights into the function of whole genomic regions or individual genes, as well as their expression and regulation, have unveiled a new understanding of the biology of imprinting and its evolutionary role in nutrition, metabolism, stress, reproduction, sleep, and the circadian clock [95]. The expression of PWS genes (e.g., the specific expression of *MAGEL2* in the hypothalamus and pituitary) further positioned the neuroendocrine function of the hypothalamus at the center of organismal homeostasis and environmental interaction, critical for individual and species success. To allow better adaptation to daily and seasonal environmental changes, the interplay between genetic and epigenetic factors evolved in the hypothalamus, which may lead to many diseases when derailed. Given *MAGEL2*′s unique tissue expression enrichment among PWS genes in both humans and mice (Figure 2) and the recent findings on its molecular/cellular role, we hypothesize that MAGEL2 evolved as a hypothalamic regulator of regulated secretion.

## 5. MAGEL2 Regulates the Recycling of Core Components of Secretory Granules in the Hypothalamus and Enables Robust Endocrine Regulation

In recent years, hormonal imbalance and its relationship with the genotype of patients have been studied as the effect of the absence or alteration of *Magel2* in mice and lately also in rats. Loss of *Magel2* in mice leads to neonatal growth retardation, obesity, altered circadian rhythm, and reduced motor activity (Figure 3B) [184,205,206]. *Magel2*-null mice also exhibit reduced fertility with females displaying prolonged and irregular estrous cycles and males showing decreased testosterone levels and reduced olfactory preference for female odors [182]. Interestingly, pheromone detection in mice is the result of a direct neural connection between the main olfactory epithelium and the hypothalamic GnRH neurons [182,206,207]. Some behavioral phenotypes were also recapitulated in the *Magel2^m+/p^^−^* rat (Figure 3B); however, there are several interspecies differences that need to be further investigated to completely understand the role of MAGEL2 and how to overcome its deficiency in patients [12]. Overall, these studies indicate that the loss of *MAGEL2* expression contributes to the reproductive deficiencies observed in PWS, as well as many other clinical features [21].

**Figure 4:**
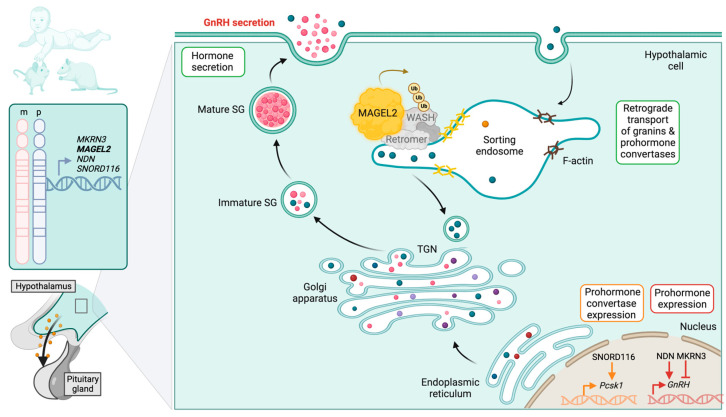
MAGEL2 and other PWS genes are maternally imprinted, paternally expressed genes that serve as tissue-specific regulators of endocrine hormones in the hypothalamus by regulating the retromer-dependent recycling pathway or expression of prohormones/secretory granule components.

The last ten years have provided several important mechanistic insights into MAGEL2 molecular and cellular functions. Like other members of the MAGE family [17], MAGEL2 is also part of a multi-subunit protein complex with the E3 ubiquitin ligase TRIM27 and the deubiquitinating enzyme ubiquitin-specific protease 7 (USP7) [42,43]. The MAGEL2–USP7–TRIM27 (MUST) complex facilitates the retromer-dependent recycling pathway through ubiquitination and activation of the actin nucleation promoter factor WASH (Figure 4) [7,42,43]. Endosomal protein recycling via the retromer complex is an essential process that facilitates the trafficking of charged membrane proteins from endosomes to the plasma membrane or the *trans*-Golgi network (TGN), thus avoiding their trafficking to and degradation in the lysosomes [208,209]. The main cargo dependent on MAGEL2 retrograde recycling are specific components of secretory granules (SGs), mainly enzymes and proteins that enable hormone processing and their condensation during maturation for long-term storage, indicating that MAGEL2 is involved in the abundance of SGs and the production of bioactive neuropeptides [73].

Regulated secretion is an essential process by which secretory cells synthesize and release cargo proteins through stimulus-dependent fusion of SGs with the plasma membrane (Figure 4). The generation of SGs from the TGN involves the aggregation of cargo proteins and their separation from unregulated secretory molecules [210]. SG maturation involves a decrease in luminal pH, membrane remodeling, condensation of granule contents, and hormone and neuropeptide maturation by endopeptidases [211]. Prohormones and neuropeptide precursors packaged into SGs are cleaved into active peptides and hormones by SG-resident prohormone convertases (PCs).

The discovery that MAGEL2 impacts regulated secretion in the hypothalamus was exciting, as neuroendocrine system deficiency is one of the core symptoms of PWS and SYS [10,73]. Chen et al. [73] quantified changes in protein levels from the hypothalamus in *Magel2* paternal truncation (*Magel2*^*m+/pΔ*^) mice and from neuronal cultures of PWS deletion induced pluripotent stem cells (iPSCs) and patient-derived dental pulp stem cells (DPSCs). Compared to control *Magel2^+/+^* littermates, the hypothalamus, and pituitary of *Magel2*^*m+/pΔ*^ mice exhibited decreases in several hormones and neuropeptides, including melanocyte-stimulating hormone (MSH), oxytocin, vasopressin, GnRH, and somatostatin (Table 1) [73]. Further, the proteomic analysis also revealed a decrease in SG components (e.g., granins and PCs) that was confirmed in the patient-derived DPSC models, and the abundance of SGs in the hypothalamus was also reduced in *Magel2^m+/p^*^*Δ*^ mice [73].

Mechanistically, the decreased abundance of various SG proteins and neuropeptides were due to impaired endosomal protein trafficking that led to their lysosomal degradation [73]. These data are directly related to the alteration of *MAGEL2*, as MAGEL2 directs the ubiquitination activity of TRIM27 and regulates endosomal traffic [7]. In particular, the retromer complex recruits the MUST complex to specialized endosomes through a direct interaction between MAGEL2 and the retromer subunit VPS35 (Figure 4). MAGEL2 then directs TRIM27 to ubiquitinate K220 of WASH, another retromer-associated protein, leading to the activation of WASH and F-actin polymerization for endosomal trafficking to occur [7,43]. Adequate endosomal trafficking by the MAGEL2-regulated WASH complex is required to prevent aberrant lysosomal degradation of SG proteins and reduced abundance of mature SGs [73]. It is of utmost interest to understand if and why MAGEL2 evolved to act as a hypothalamic-specific activator of WASH and F-actin polymerization, given that WASH protein and retromer recycling occurs ubiquitously and even in lower eukaryotes [212,213].

*MAGEL2* loss is associated with hyperphagia through impaired function of the hypothalamic arcuate nucleus, which regulates food intake and body weight through the complex interactions of neuropeptide Y (NPY), agouti-related peptide (AgRP), proopiomelanocortin (POMC), and leptin [21,214]. While POMC reduces food intake, the interaction between NPY and AgRP stimulates food intake [21]. Interestingly, MAGEL2 in complex with Necdin, another E3 ligase RNF41, and deubiquitinating enzyme USP8 regulates recycling of the leptin receptor through the endosomal sorting complexes required for transport (ESCRT)-0 complex [45,215]. Additionally, the loss of *Magel2* in mice abolishes leptin-mediated depolarization of POMC hypothalamic neurons [21,216,217]. This neural defect leads to less repression of food intake and uncontrolled leptin-regulated fat storage; thus, the dysregulation of leptin receptor activity upon loss of *Magel2* may be the underlying cellular mechanism for obesity in PWS [21,45].

Retromer is also important for recycling membrane proteins like hormone or neuropeptide receptors and nutrient transporters; however, the role of MAGEL2-dependent retromer recycling in hypothalamic receptor expression still awaits exploration [218].

## 6. Conclusions

In summary, the molecular functions of PWS genes, particularly the two MAGE genes *NDN* and *MAGEL2*, indicate that they evolved to finetune the diverse neuroendocrine functions in the hypothalamus. Interestingly, several PWS genes function as or in concert with ubiquitin ligases, highlighting ubiquitination as an important posttranslational modification that may promote stress adaptation by the hypothalamus. Furthermore, MAGEL2 has emerged as a tissue-specific regulator of the core secretory machinery through the recycling of the core secretory granule components, which can be reused in several rounds of hormone secretion. Thus, protein recycling represents an evolutionary adaptation and advantage, and finetuning of this process enables better neuroendocrine control over adaptation to diverse environmental cues. Several interesting questions remain open.

For instance, our understanding of the function of PWS genes within the placenta and their potential link to brain development, such as embryonic hypothalamus development and subsequent lifelong physiological roles, remains limited. Notably, the placenta serves as a dynamic endocrine organ, pivotal in shaping pregnancy advancement and maternal metabolic, endocrine, and immune system adjustments [219]. It will be intriguing to unveil the contributions of *MAGEL2* and other PWS genes in placental endocrine activities.

Moreover, while certain behavioral traits are common between mouse and rat models (Figure 3), disparities across species warrant in-depth exploration to comprehensively fathom the significance of MAGEL2 functions and strategies to address its insufficiency in patients. Another point of interest lies in the retromer’s role in recycling membrane proteins like hormone receptors, neuropeptide receptors, and nutrient transporters. The involvement of MAGEL2-dependent retromer recycling in the expression of hypothalamic receptors remains a topic awaiting further investigation [42,209]

Our understanding of the transcriptional and posttranscriptional regulation of PWS genes, including *MAGEL2*, is limited. Furthermore, the effects of complete *MAGEL2* deletions on the expression of other PWS genes, and whether there exist regulatory elements that could partially compensate for *MAGEL2*’s functional loss, remain unexplored.

All these questions will be best addressed by investigating the role of *MAGEL2* and other genes in the imprinted PWS region from an evolutionary, comparative, and molecular perspective. These may also lead to a better understanding of the recycling of secretory granule components and the regulation of hormone and neuropeptide secretion, and above all, to novel hints on how we could therapeutically overcome the deficiencies in patients and improve their quality of life.

## Figures and Tables

**Table 1:** Overview of the hormonal and neuropeptide imbalances in PWS and SYS patients and mouse models.

Name	Tissue	Expression in PWS		References
		Patients	Mouse (Model)	
Follicle-stimulating hormone (FSH)	Anterior pituitary	↑ (variable in male and female)	--	[49,134]
Growth hormone (GH)	Anterior pituitary	↓	↓ (*Magel2^m+/p^*^*Δ*^)	[10,73,135]
Luteinizing hormone (LH)	Anterior pituitary	↑ (variable in male and female)	↓ (*Magel2^m+/p^*^*Δ*^)	[54,73,134,136]
Melanocyte-stimulating hormone (*α*-MSH, *β*-MSH)	Anterior pituitary	↓	↓ (*Magel2^m+/p^^*Δ*^*)	[73,137,138]
Prolactin (PRL)	Anterior pituitary	--	↓ (*Magel2^m+/p^*^*Δ*^)	[73]
Proopiomelanocortin (POMC) *	Anterior pituitary	↓ in CNS	↑ (PWS IC deletion)	[138,139,140]
Cortisol/Corticosterone	Adrenal cortex	--	↑ (TgPWS)	[141]
Chromogranins (A, B)	Adrenal medulla and pancreas	↓	↓ (*Magel2^m+^*/*^p^*^*Δ*^*)*	[73]
Proenkephalin (PENK)	CNS and adrenal medulla	--	↓ (*Magel2^m+^*/*^p^*^*Δ*^)	[65,73]
Nesfatin-1	CNS, adipose, gonads, stomach, pancreas, and liver	↑	--	[142]
Brain-derived neurotrophic factor (BDNF)	CNS, lungs, heart, spleen, GI tract, and liver	↓ and ↑ are reported	--	[137,138,139,143]
Insulin growth factor binding protein 7 (IGFBP7)	CNS, GI tract, liver, kidney, adrenal cortex, lung, testis, and ovary	↑ in neuronal cells	↑ (PWScr*^m+/p−^*)	[144]
Peptide YY (PYY)	GI tract	↓ and ↑ are reported	--	[145,146]
Substance P (SP)	GI tract	↑	--	[147]
Adrenocorticotropic hormone (ACTH)	Anterior pituitary	↓; no change	--	[148,149,150]
Agouti-related protein (AgRP)	Hypothalamus (arcuate nucleus)	↓ and no change are reported	↓ and ↑ are reported (*Snord116^m+/p−^*)	[14,137,138]
β-endorphin (BE)	Hypothalamus and anterior pituitary	↑	--	[147]
Galanin	Hypothalamus, pituitary, and GI tract	↓	↓ (*Magel2^m+^*/*^p^*^*Δ*^)	[73]
Gonadotropin-releasing hormone (GnRH)	Hypothalamus	↓	↓ (*Magel2^m+^*/*^p^*^*Δ*^) and no change (*Mkrn3^m+/p−^*)	[36,49,73]
Kisspeptin	Hypothalamus (arcuate nucleus)	--	No change (*Mkrn3^m+/p−^*)	[36]
Melanin-concentrating hormone (MCH)	Hypothalamus	--	No change (PWScr*^m+/p−^*)	[151,152]
Neurokinin B (NKB)	Hypothalamus (arcuate nucleus)	--	↑ (*Mkrn3^m+/p−^*)	[36]
Neuropeptide Y (NPY)	Hypothalamus (arcuate nucleus)	↑; ↓ in CNS	↑ (*Snord116^m+/p−^*)	[14,138]
Orexin/hypocretin	Hypothalamus	↓ in CSF; ↑ in plasma	↓ (*Magel2^m+/p^*^*Δ*^; PWScr*^m+^*/*^p−^*)	[65,152,153,154,155,156]
Oxytocin (OXT)	Hypothalamus (paraventricular and supraoptic nuclei) and posterior pituitary	↓ and ↑ are reported	↓ (*Magel^2m+^*/*^p^*^*Δ*^)	[73,138,157,158]
Somatostatin (SST)	Hypothalamus and GI tract	↓	↓ (*Magel2^m+^*/*^p^*^*Δ*^)	[65,73,141]
Thyrotropin-releasing hormone (TRH)	Hypothalamus	--	↓ (*Magel2^m+^*/*^p^*^*Δ*^)	[73,159,160]
Vasopressin (AVP)	Hypothalamus	↓	↓ (*Magel2^m+^*/*^p^*^*Δ*^)	[65,73]
Glucagon	Pancreas (α-cells)	--	↓ (TgPWS)	[141]
Amylin/Islet amyloid polypeptide (IAPP)	Pancreas (β-cells)	↓	--	[10,161]
Insulin	Pancreas (β-cells)	↓ and ↑ are reported	↓ (TgPWS)	[10,65,138,146]
Obestatin	Stomach	↑ and no change are reported	--	[162,163]
Ghrelin	Stomach, hypothalamus (arcuate and paraventricular nuclei), pituitary, lung, adrenal cortex, and pancreas	↑	↑ (TgPWS; *Snord116^m+/p−^*)	[14,138,141,163,164,165]
Testosterone	Testis	↓ (male)	--	[49,134,136]
Anti-Mullerian hormone (AMH)/Mullerian inhibiting hormone (MIH)	Testis and ovary	↓ (female)	--	[49,166]
Triiodothyronine (T3)	Thyroid gland	No change	--	[159,167,168,169]
Thyroxine (T4)	Thyroid gland	↓ (infants)	--	[159,169,170]
Adiponectin	White adipose	↑ and no change are reported	--	[142,146,171,172,173]
Resistin	White adipose	↑ and no change are reported	--	[174,175]
Leptin	White adipose	↑ and no change are reported	No change (*Snord116^m+/p−^*)	[74,142,143,176,177,178]
Spexin (Spx)	White adipose, hypothalamus, adrenal gland, pancreas, thyroid, and GI tract	↓	--	[142,179]

* Processing of POMC produces 10 peptides: β-endorphin; corticotropin (adrenocorticotropic hormone (ACTH)); N-terminal peptide of proopiomelanocortin (NPP or pro-γ-MSH); α-, β-, and γ-melanotropin (α-, β-, and γ-MSH); corticotropin-like intermediate peptide (CLIP); β- and γ-lipotropin (β- and γ-LPH); and (Met)enkephalin.

## References

[1] Cassidy S.B., Schwartz S., Miller J.L., Driscoll D.J. (2012). Prader-Willi syndrome. Genet. Med..

[2] Butler M.G., Hartin S.N., Hossain W.A., Manzardo A.M., Kimonis V., Dykens E., Gold J.A., Kim S.J., Weisensel N., Tamura R. (2019). Molecular genetic classification in Prader-Willi syndrome: A multisite cohort study. J. Med. Genet..

[3] Nicholls R.D., Knepper J.L. (2001). Genome organization, function, and imprinting in Prader-Willi and Angelman syndromes. Annu. Rev. Genom. Hum. Genet..

[4] Butler M.G. (2023). Prader-Willi Syndrome and Chromosome 15q11.2 BP1-BP2 Region: A Review. Int. J. Mol. Sci..

[5] Marbach F., Elgizouli M., Rech M., Beygo J., Erger F., Velmans C., Stumpel C., Stegmann A.P.A., Beck-Wodl S., Gillessen-Kaesbach G. (2020). The adult phenotype of Schaaf-Yang syndrome. Orphanet J. Rare Dis..

[6] Driscoll D.J., Miller J.L., Cassidy S.B., Adam M.P., Mirzaa G.M., Pagon R.A., Wallace S.E., Bean L.J.H., Gripp K.W., Amemiya A. (1993). Prader-Willi Syndrome. GeneReviews ((R)).

[7] Fon Tacer K., Potts P.R. (2017). Cellular and disease functions of the Prader-Willi Syndrome gene MAGEL2. Biochem. J..

[8] Schaaf C.P., Gonzalez-Garay M.L., Xia F., Potocki L., Gripp K.W., Zhang B., Peters B.A., McElwain M.A., Drmanac R., Beaudet A.L. (2013). Truncating mutations of MAGEL2 cause Prader-Willi phenotypes and autism. Nat. Genet..

[9] Fountain M.D., Aten E., Cho M.T., Juusola J., Walkiewicz M.A., Ray J.W., Xia F., Yang Y., Graham B.H., Bacino C.A. (2017). The phenotypic spectrum of Schaaf-Yang syndrome: 18 new affected individuals from 14 families. Genet. Med..

[10] Koppes E.A., Johnson M.A., Moresco J.J., Luppi P., Lewis D.W., Stolz D.B., Diedrich J.K., Yates J.R., Wek R.C., Watkins S.C. (2023). Insulin secretion deficits in a Prader-Willi syndrome beta-cell model are associated with a concerted downregulation of multiple endoplasmic reticulum chaperones. PLoS Genet..

[11] Kummerfeld D.M., Raabe C.A., Brosius J., Mo D., Skryabin B.V., Rozhdestvensky T.S. (2021). A Comprehensive Review of Genetically Engineered Mouse Models for Prader-Willi Syndrome Research. Int. J. Mol. Sci..

[12] Reznik D.L., Yang M.V., de la Haza P.A., Jain A., Spanjaard M., Theiss S., Schaaf C.P., Malovannaya A., Strong T.V., Veeraragavan S. (2023). Magel2 truncation alters select behavioral and physiological outcomes in a rat model of Schaaf-Yang syndrome. Dis. Model. Mech..

[13] Meader B.N., Albano A., Sekizkardes H., Delaney A. (2020). Heterozygous Deletions in MKRN3 Cause Central Precocious Puberty Without Prader-Willi Syndrome. J. Clin. Endocrinol. Metab..

[14] Burnett L.C., LeDuc C.A., Sulsona C.R., Paull D., Rausch R., Eddiry S., Carli J.F., Morabito M.V., Skowronski A.A., Hubner G. (2017). Deficiency in prohormone convertase PC1 impairs prohormone processing in Prader-Willi syndrome. J. Clin. Investig..

[15] Fon Tacer K., Montoya M.C., Oatley M.J., Lord T., Oatley J.M., Klein J., Ravichandran R., Tillman H., Kim M., Connelly J.P. (2019). MAGE cancer-testis antigens protect the mammalian germline under environmental stress. Sci. Adv..

[16] Lee A.K., Klein J., Fon Tacer K., Lord T., Oatley M.J., Oatley J.M., Porter S.N., Pruett-Miller S.M., Tikhonova E.B., Karamyshev A.L. (2020). Translational Repression of G3BP in Cancer and Germ Cells Suppresses Stress Granules and Enhances Stress Tolerance. Mol. Cell.

[17] Florke Gee R.R., Chen H., Lee A.K., Daly C.A., Wilander B.A., Fon Tacer K., Potts P.R. (2020). Emerging roles of the MAGE protein family in stress response pathways. J. Biol. Chem..

[18] Su A.I., Cooke M.P., Ching K.A., Hakak Y., Walker J.R., Wiltshire T., Orth A.P., Vega R.G., Sapinoso L.M., Moqrich A. (2002). Large-scale analysis of the human and mouse transcriptomes. Proc. Natl. Acad. Sci. USA.

[19] Su A.I., Wiltshire T., Batalov S., Lapp H., Ching K.A., Block D., Zhang J., Soden R., Hayakawa M., Kreiman G. (2004). A gene atlas of the mouse and human protein-encoding transcriptomes. Proc. Natl. Acad. Sci. USA.

[20] Cavaille J., Buiting K., Kiefmann M., Lalande M., Brannan C.I., Horsthemke B., Bachellerie J.P., Brosius J., Huttenhofer A. (2000). Identification of brain-specific and imprinted small nucleolar RNA genes exhibiting an unusual genomic organization. Proc. Natl. Acad. Sci. USA.

[21] Costa R.A., Ferreira I.R., Cintra H.A., Gomes L.H.F., Guida L.D.C. (2019). Genotype-Phenotype Relationships and Endocrine Findings in Prader-Willi Syndrome. Front. Endocrinol..

[22] Keshavarz M., Savriama Y., Refki P., Reeves R.G., Tautz D. (2021). Natural copy number variation of tandemly repeated regulatory SNORD RNAs leads to individual phenotypic differences in mice. Mol. Ecol..

[23] Kanber D., Giltay J., Wieczorek D., Zogel C., Hochstenbach R., Caliebe A., Kuechler A., Horsthemke B., Buiting K. (2009). A paternal deletion of MKRN3, MAGEL2 and NDN does not result in Prader-Willi syndrome. Eur. J. Hum. Genet..

[24] Bervini S., Herzog H. (2013). Mouse models of Prader-Willi Syndrome: A systematic review. Front. Neuroendocr..

[25] Gray T.A., Saitoh S., Nicholls R.D. (1999). An imprinted, mammalian bicistronic transcript encodes two independent proteins. Proc. Natl. Acad. Sci. USA.

[26] Resnick J.L., Nicholls R.D., Wevrick R., Prader-Willi Syndrome Animal Models Working G. (2013). Recommendations for the investigation of animal models of Prader-Willi syndrome. Mamm. Genome.

[27] Jong M.T., Gray T.A., Ji Y., Glenn C.C., Saitoh S., Driscoll D.J., Nicholls R.D. (1999). A novel imprinted gene, encoding a RING zinc-finger protein, and overlapping antisense transcript in the Prader-Willi syndrome critical region. Hum. Mol. Genet..

[28] Jong M.T., Carey A.H., Caldwell K.A., Lau M.H., Handel M.A., Driscoll D.J., Stewart C.L., Rinchik E.M., Nicholls R.D. (1999). Imprinting of a RING zinc-finger encoding gene in the mouse chromosome region homologous to the Prader-Willi syndrome genetic region. Hum. Mol. Genet..

[29] Abreu A.P., Toro C.A., Song Y.B., Navarro V.M., Bosch M.A., Eren A., Liang J.N., Carroll R.S., Latronico A.C., Ronnekleiv O.K. (2020). MKRN3 inhibits the reproductive axis through actions in kisspeptin-expressing neurons. J. Clin. Investig..

[30] Gray T.A., Hernandez L., Carey A.H., Schaldach M.A., Smithwick M.J., Rus K., Marshall Graves J.A., Stewart C.L., Nicholls R.D. (2000). The ancient source of a distinct gene family encoding proteins featuring RING and C(3)H zinc-finger motifs with abundant expression in developing brain and nervous system. Genomics.

[31] Macedo D.B., Abreu A.P., Reis A.C., Montenegro L.R., Dauber A., Beneduzzi D., Cukier P., Silveira L.F., Teles M.G., Carroll R.S. (2014). Central precocious puberty that appears to be sporadic caused by paternally inherited mutations in the imprinted gene makorin ring finger 3. J. Clin. Endocrinol. Metab..

[32] Ludwig N.G., Radaeli R.F., Silva M.M., Romero C.M., Carrilho A.J., Bessa D., Macedo D.B., Oliveira M.L., Latronico A.C., Mazzuco T.L. (2016). A boy with Prader-Willi syndrome: Unmasking precocious puberty during growth hormone replacement therapy. Arch. Endocrinol. Metab..

[33] Lee H.S., Hwang J.S. (2013). Central precocious puberty in a girl with Prader-Willi syndrome. J. Pediatr. Endocrinol. Metab..

[34] Li C., Han T., Li Q., Zhang M., Guo R., Yang Y., Lu W., Li Z., Peng C., Wu P. (2021). MKRN3-mediated ubiquitination of Poly(A)-binding proteins modulates the stability and translation of GNRH1 mRNA in mammalian puberty. Nucleic Acids Res..

[35] Li C., Lu W., Yang L., Li Z., Zhou X., Guo R., Wang J., Wu Z., Dong Z., Ning G. (2020). MKRN3 regulates the epigenetic switch of mammalian puberty via ubiquitination of MBD3. Natl. Sci. Rev..

[36] Naule L., Mancini A., Pereira S.A., Gassaway B.M., Lydeard J.R., Magnotto J.C., Kim H.K., Liang J., Matos C., Gygi S.P. (2023). MKRN3 inhibits puberty onset via interaction with IGF2BP1 and regulation of hypothalamic plasticity. JCI Insight.

[37] Liu H., Kong X., Chen F. (2017). Mkrn3 functions as a novel ubiquitin E3 ligase to inhibit Nptx1 during puberty initiation. Oncotarget.

[38] Yellapragada V., Liu X., Lund C., Kansakoski J., Pulli K., Vuoristo S., Lundin K., Tuuri T., Varjosalo M., Raivio T. (2019). MKRN3 Interacts With Several Proteins Implicated in Puberty Timing but Does Not Influence GNRH1 Expression. Front. Endocrinol..

[39] Valadares L.P., Meireles C.G., De Toledo I.P., de Oliveira R.S., de Castro L.C.G., Abreu A.P., Carroll R.S., Latronico A.C., Kaiser U.B., Guerra E.N.S. (2019). MKRN3 Mutations in Central Precocious Puberty: A Systematic Review and Meta-Analysis. J. Endocr. Soc..

[40] Abreu A.P., Dauber A., Macedo D.B., Noel S.D., Brito V.N., Gill J.C., Cukier P., Thompson I.R., Navarro V.M., Gagliardi P.C. (2013). Central precocious puberty caused by mutations in the imprinted gene MKRN3. N. Engl. J. Med..

[41] Doyle J.M., Gao J., Wang J., Yang M., Potts P.R. (2010). MAGE-RING protein complexes comprise a family of E3 ubiquitin ligases. Mol. Cell.

[42] Hao Y.H., Fountain M.D., Fon Tacer K., Xia F., Bi W., Kang S.H., Patel A., Rosenfeld J.A., Le Caignec C., Isidor B. (2015). USP7 Acts as a Molecular Rheostat to Promote WASH-Dependent Endosomal Protein Recycling and Is Mutated in a Human Neurodevelopmental Disorder. Mol. Cell.

[43] Hao Y.H., Doyle J.M., Ramanathan S., Gomez T.S., Jia D., Xu M., Chen Z.J., Billadeau D.D., Rosen M.K., Potts P.R. (2013). Regulation of WASH-dependent actin polymerization and protein trafficking by ubiquitination. Cell.

[44] Schaller F., Watrin F., Sturny R., Massacrier A., Szepetowski P., Muscatelli F. (2010). A single postnatal injection of oxytocin rescues the lethal feeding behaviour in mouse newborns deficient for the imprinted Magel2 gene. Hum. Mol. Genet..

[45] Wijesuriya T.M., De Ceuninck L., Masschaele D., Sanderson M.R., Carias K.V., Tavernier J., Wevrick R. (2017). The Prader-Willi syndrome proteins MAGEL2 and necdin regulate leptin receptor cell surface abundance through ubiquitination pathways. Hum. Mol. Genet..

[46] Lu R., Dong Y., Li J.D. (2020). Necdin regulates BMAL1 stability and circadian clock through SGT1-HSP90 chaperone machinery. Nucleic Acids Res..

[47] Kuwajima T., Nishimura I., Yoshikawa K. (2006). Necdin promotes GABAergic neuron differentiation in cooperation with Dlx homeodomain proteins. J. Neurosci..

[48] Miller N.L., Wevrick R., Mellon P.L. (2009). Necdin, a Prader-Willi syndrome candidate gene, regulates gonadotropin-releasing hormone neurons during development. Hum. Mol. Genet..

[49] Napolitano L., Barone B., Morra S., Celentano G., La Rocca R., Capece M., Morgera V., Turco C., Caputo V.F., Spena G. (2021). Hypogonadism in Patients with Prader Willi Syndrome: A Narrative Review. Int. J. Mol. Sci..

[50] Wu R.N., Hung W.C., Chen C.T., Tsai L.P., Lai W.S., Min M.Y., Wong S.B. (2020). Firing activity of locus coeruleus noradrenergic neurons decreases in necdin-deficient mice, an animal model of Prader-Willi syndrome. J. Neurodev. Disord..

[51] Gerard M., Hernandez L., Wevrick R., Stewart C.L. (1999). Disruption of the mouse necdin gene results in early post-natal lethality. Nat. Genet..

[52] Rieusset A., Schaller F., Unmehopa U., Matarazzo V., Watrin F., Linke M., Georges B., Bischof J., Dijkstra F., Bloemsma M. (2013). Stochastic loss of silencing of the imprinted Ndn/NDN allele, in a mouse model and humans with prader-willi syndrome, has functional consequences. PLoS Genet..

[53] Watrin F., Roeckel N., Lacroix L., Mignon C., Mattei M.G., Disteche C., Muscatelli F. (1997). The mouse Necdin gene is expressed from the paternal allele only and lies in the 7C region of the mouse chromosome 7, a region of conserved synteny to the human Prader-Willi syndrome region. Eur. J. Hum. Genet..

[54] Muscatelli F., Abrous D.N., Massacrier A., Boccaccio I., Le Moal M., Cau P., Cremer H. (2000). Disruption of the mouse Necdin gene results in hypothalamic and behavioral alterations reminiscent of the human Prader-Willi syndrome. Hum. Mol. Genet..

[55] Matarazzo V., Caccialupi L., Schaller F., Shvarev Y., Kourdougli N., Bertoni A., Menuet C., Voituron N., Deneris E., Gaspar P. (2017). Necdin shapes serotonergic development and SERT activity modulating breathing in a mouse model for Prader-Willi syndrome. Elife.

[56] Farber C., Gross S., Neesen J., Buiting K., Horsthemke B. (2000). Identification of a testis-specific gene (C15orf2) in the Prader-Willi syndrome region on chromosome 15. Genomics.

[57] Neumann L.C., Markaki Y., Mladenov E., Hoffmann D., Buiting K., Horsthemke B. (2012). The imprinted NPAP1/C15orf2 gene in the Prader-Willi syndrome region encodes a nuclear pore complex associated protein. Hum. Mol. Genet..

[58] Neumann L.C., Feiner N., Meyer A., Buiting K., Horsthemke B. (2014). The imprinted NPAP1 gene in the Prader-Willi syndrome region belongs to a POM121-related family of retrogenes. Genome Biol. Evol..

[59] Jonkers J., van Amerongen R., van der Valk M., Robanus-Maandag E., Molenaar M., Destree O., Berns A. (1999). In vivo analysis of Frat1 deficiency suggests compensatory activity of Frat3. Mech. Dev..

[60] Glenn C.C., Saitoh S., Jong M.T., Filbrandt M.M., Surti U., Driscoll D.J., Nicholls R.D. (1996). Gene structure, DNA methylation, and imprinted expression of the human SNRPN gene. Am. J. Hum. Genet..

[61] Cheon C.K. (2016). Genetics of Prader-Willi syndrome and Prader-Will-Like syndrome. Ann. Pediatr. Endocrinol. Metab..

[62] Carias K.V., Wevrick R. (2019). Preclinical Testing in Translational Animal Models of Prader-Willi Syndrome: Overview and Gap Analysis. Mol. Ther. Methods Clin. Dev..

[63] Cao Y., AlHumaidi S.S., Faqeih E.A., Pitel B.A., Lundquist P., Aypar U. (2017). A novel deletion of SNURF/SNRPN exon 1 in a patient with Prader-Willi-like phenotype. Eur. J. Med. Genet..

[64] Huang Y., Grand K., Kimonis V., Butler M.G., Jain S., Huang A.Y., Martinez-Agosto J.A., Nelson S.F., Sanchez-Lara P.A. (2022). Mosaic de novo SNRPN gene variant associated with Prader-Willi syndrome. J. Med. Genet..

[65] Basak S., Basak A. (2022). Proteins and proteases of Prader-Willi syndrome: A comprehensive review and perspectives. Biosci. Rep..

[66] Bieth E., Eddiry S., Gaston V., Lorenzini F., Buffet A., Conte Auriol F., Molinas C., Cailley D., Rooryck C., Arveiler B. (2015). Highly restricted deletion of the SNORD116 region is implicated in Prader-Willi Syndrome. Eur. J. Hum. Genet..

[67] Duker A.L., Ballif B.C., Bawle E.V., Person R.E., Mahadevan S., Alliman S., Thompson R., Traylor R., Bejjani B.A., Shaffer L.G. (2010). Paternally inherited microdeletion at 15q11.2 confirms a significant role for the SNORD116 C/D box snoRNA cluster in Prader-Willi syndrome. Eur. J. Hum. Genet..

[68] Sahoo T., del Gaudio D., German J.R., Shinawi M., Peters S.U., Person R.E., Garnica A., Cheung S.W., Beaudet A.L. (2008). Prader-Willi phenotype caused by paternal deficiency for the HBII-85 C/D box small nucleolar RNA cluster. Nat. Genet..

[69] de Smith A.J., Purmann C., Walters R.G., Ellis R.J., Holder S.E., Van Haelst M.M., Brady A.F., Fairbrother U.L., Dattani M., Keogh J.M. (2009). A deletion of the HBII-85 class of small nucleolar RNAs (snoRNAs) is associated with hyperphagia, obesity and hypogonadism. Hum. Mol. Genet..

[70] Kim S.J., Miller J.L., Kuipers P.J., German J.R., Beaudet A.L., Sahoo T., Driscoll D.J. (2012). Unique and atypical deletions in Prader-Willi syndrome reveal distinct phenotypes. Eur. J. Hum. Genet..

[71] Runte M., Huttenhofer A., Gross S., Kiefmann M., Horsthemke B., Buiting K. (2001). The IC-SNURF-SNRPN transcript serves as a host for multiple small nucleolar RNA species and as an antisense RNA for UBE3A. Hum. Mol. Genet..

[72] Qi Y., Purtell L., Fu M., Lee N.J., Aepler J., Zhang L., Loh K., Enriquez R.F., Baldock P.A., Zolotukhin S. (2016). Snord116 is critical in the regulation of food intake and body weight. Sci. Rep..

[73] Chen H., Victor A.K., Klein J., Tacer K.F., Tai D.J., de Esch C., Nuttle A., Temirov J., Burnett L.C., Rosenbaum M. (2020). Loss of MAGEL2 in Prader-Willi syndrome leads to decreased secretory granule and neuropeptide production. JCI Insight.

[74] Polex-Wolf J., Lam B.Y., Larder R., Tadross J., Rimmington D., Bosch F., Cenzano V.J., Ayuso E., Ma M.K., Rainbow K. (2018). Hypothalamic loss of Snord116 recapitulates the hyperphagia of Prader-Willi syndrome. J. Clin. Investig..

[75] Polvora-Brandao D., Joaquim M., Godinho I., Aprile D., Alvaro A.R., Onofre I., Raposo A.C., Pereira de Almeida L., Duarte S.T., da Rocha S.T. (2018). Loss of hierarchical imprinting regulation at the Prader-Willi/Angelman syndrome locus in human iPSCs. Hum. Mol. Genet..

[76] Salminen I.I., Crespi B.J., Mokkonen M. (2019). Baby food and bedtime: Evidence for opposite phenotypes from different genetic and epigenetic alterations in Prader-Willi and Angelman syndromes. SAGE Open Med..

[77] Barlow D.P. (2011). Genomic imprinting: A mammalian epigenetic discovery model. Annu. Rev. Genet..

[78] Chung M.S., Langouet M., Chamberlain S.J., Carmichael G.G. (2020). Prader-Willi syndrome: Reflections on seminal studies and future therapies. Open Biol..

[79] Horsthemke B., Wagstaff J. (2008). Mechanisms of imprinting of the Prader-Willi/Angelman region. Am. J. Med. Genet. A.

[80] Buiting K., Lich C., Cottrell S., Barnicoat A., Horsthemke B. (1999). A 5-kb imprinting center deletion in a family with Angelman syndrome reduces the shortest region of deletion overlap to 880 bp. Hum. Genet..

[81] Shemer R., Hershko A.Y., Perk J., Mostoslavsky R., Tsuberi B., Cedar H., Buiting K., Razin A. (2000). The imprinting box of the Prader-Willi/Angelman syndrome domain. Nat. Genet..

[82] Saitoh S., Wada T. (2000). Parent-of-Origin Specific Histone Acetylation and Reactivation of a Key Imprinted Gene Locus in Prader-Willi Syndrome. Am. J. Hum. Genet..

[83] Schweizer J., Zynger D., Francke U. (1999). In vivo Nuclease Hypersensitivity Studies Reveal Multiple Sites of Parental Origin-Dependent Differential Chromatin Conformation in the 150 Kb SNRPN Transcription Unit. Hum. Mol. Genet..

[84] Perk J., Makedonski K., Lande L., Cedar H., Razin A., Shemer R. (2002). The imprinting mechanism of the Prader-Willi/Angelman regional control center. EMBO J..

[85] Brant J.O., Riva A., Resnick J.L., Yang T.P. (2014). Influence of the Prader-Willi syndrome imprinting center on the DNA methylation landscape in the mouse brain. Epigenetics.

[86] El-Maarri O., Buiting K., Peery E.G., Kroisel P.M., Balaban B., Wagner K., Urman B., Heyd J., Lich C., Brannan C.I. (2001). Maternal methylation imprints on human chromosome 15 are established during or after fertilization. Nat. Genet..

[87] Huntriss J., Hinkins M., Oliver B., Harris S.E., Beazley J.C., Rutherford A.J., Gosden R.G., Lanzendorf S.E., Picton H.M. (2004). Expression of mRNAs for DNA methyltransferases and methyl-CpG-binding proteins in the human female germ line, preimplantation embryos, and embryonic stem cells. Mol. Reprod. Dev..

[88] Kantor B., Kaufman Y., Makedonski K., Razin A., Shemer R. (2004). Establishing the epigenetic status of the Prader–Willi/Angelman imprinting center in the gametes and embryo. Hum. Mol. Genet..

[89] DuBose A.J., Smith E.Y., Johnstone K.A., Resnick J.L. (2012). Temporal and developmental requirements for the Prader-Willi imprinting center. Proc. Natl. Acad. Sci. USA.

[90] Jiang C., Yang Y., Huang C., Whitelaw B. (2014). Promoter characterization and functional association with placenta of porcine MAGEL2. Gene.

[91] Renfree M.B., Hore T.A., Shaw G., Graves J.A., Pask A.J. (2009). Evolution of genomic imprinting: Insights from marsupials and monotremes. Annu. Rev. Genom. Hum. Genet..

[92] Plasschaert R.N., Bartolomei M.S. (2014). Genomic imprinting in development, growth, behavior and stem cells. Development.

[93] Peters J. (2014). The role of genomic imprinting in biology and disease: An expanding view. Nat. Rev. Genet..

[94] Moore T., Haig D. (1991). Genomic imprinting in mammalian development: A parental tug-of-war. Trends Genet..

[95] Tucci V., Isles A.R., Kelsey G., Ferguson-Smith A.C., Erice Imprinting G. (2019). Genomic Imprinting and Physiological Processes in Mammals. Cell.

[96] Gregg C., Zhang J., Weissbourd B., Luo S., Schroth G.P., Haig D., Dulac C. (2010). High-resolution analysis of parent-of-origin allelic expression in the mouse brain. Science.

[97] Higgs M.J., Hill M.J., John R.M., Isles A.R. (2022). Systematic investigation of imprinted gene expression and enrichment in the mouse brain explored at single-cell resolution. BMC Genom..

[98] Broad K.D., Keverne E.B. (2011). Placental protection of the fetal brain during short-term food deprivation. Proc. Natl. Acad. Sci. USA.

[99] Davies W., Lynn P.M., Relkovic D., Wilkinson L.S. (2008). Imprinted genes and neuroendocrine function. Front. Neuroendocr..

[100] Xie Y., Dorsky R.I. (2017). Development of the hypothalamus: Conservation, modification and innovation. Development.

[101] Butler M.G. (1990). Prader-Willi syndrome: Current understanding of cause and diagnosis. Am. J. Med. Genet..

[102] Miller J.L., Lynn C.H., Driscoll D.C., Goldstone A.P., Gold J.A., Kimonis V., Dykens E., Butler M.G., Shuster J.J., Driscoll D.J. (2011). Nutritional phases in Prader-Willi syndrome. Am. J. Med. Genet. A.

[103] Butler M.G., Miller J.L., Forster J.L. (2019). Prader-Willi Syndrome-Clinical Genetics, Diagnosis and Treatment Approaches: An Update. Curr. Pediatr. Rev..

[104] Butler J.V., Whittington J.E., Holland A.J., Boer H., Clarke D., Webb T. (2002). Prevalence of, and risk factors for, physical ill-health in people with Prader-Willi syndrome: A population-based study. Dev. Med. Child Neurol..

[105] Yang A., Kim J., Cho S.Y., Jin D.K. (2017). Prevalence and risk factors for type 2 diabetes mellitus with Prader-Willi syndrome: A single center experience. Orphanet J. Rare Dis..

[106] Angulo M.A., Butler M.G., Cataletto M.E. (2015). Prader-Willi syndrome: A review of clinical, genetic, and endocrine findings. J. Endocrinol. Investig..

[107] Teke Kısa P., Güzel O., Arslan N., Demir K. (2023). Positive effects of ketogenic diet on weight control in children with obesity due to Prader–Willi syndrome. Clin. Endocrinol..

[108] Louveau C., Turtuluci M.-C., Consoli A., Poitou C., Coupaye M., Krebs M.-O., Chaumette B., Iftimovici A. (2023). Prader–Willi syndrome: Symptoms and topiramate response in light of genetics. Front. Neurosci..

[109] Bhargava S.A., Putnam P.E., Kocoshis S.A., Rowe M., Hanchett J.M. (1996). Rectal bleeding in Prader-Willi syndrome. Pediatrics.

[110] Boer H., Holland A., Whittington J., Butler J., Webb T., Clarke D. (2002). Psychotic illness in people with Prader Willi syndrome due to chromosome 15 maternal uniparental disomy. Lancet.

[111] Soni S., Whittington J., Holland A.J., Webb T., Maina E., Boer H., Clarke D. (2007). The course and outcome of psychiatric illness in people with Prader-Willi syndrome: Implications for management and treatment. J. Intellect. Disabil. Res..

[112] Soni S., Whittington J., Holland A.J., Webb T., Maina E.N., Boer H., Clarke D. (2008). The phenomenology and diagnosis of psychiatric illness in people with Prader-Willi syndrome. Psychol. Med..

[113] Bennett J.A., Germani T., Haqq A.M., Zwaigenbaum L. (2015). Autism spectrum disorder in Prader-Willi syndrome: A systematic review. Am. J. Med. Genet. A.

[114] Butler M.G., Manzardo A.M., Heinemann J., Loker C., Loker J. (2017). Causes of death in Prader-Willi syndrome: Prader-Willi Syndrome Association (USA) 40-year mortality survey. Genet. Med..

[115] McCandless S.E., Marissa S., Yin D., Yeh M., Czado S., Aghsaei S., Li J.W., Francis K., Hadker N., Stafford D.E.J. (2020). SUN-604 U.S. Prevalence & Mortality of Prader-Willi Syndrome: A Population-Based Study of Medical Claims. J. Endocr. Soc..

[116] Manzardo A.M., Loker J., Heinemann J., Loker C., Butler M.G. (2018). Survival trends from the Prader-Willi Syndrome Association (USA) 40-year mortality survey. Genet. Med..

[117] Erhardt E., Molnar D. (2022). Prader-Willi Syndrome: Possibilities of Weight Gain Prevention and Treatment. Nutrients.

[118] Cassidy S.B., Driscoll D.J. (2009). Prader-Willi syndrome. Eur. J. Hum. Genet..

[119] Grugni G., Sartorio A., Crino A. (2016). Growth hormone therapy for Prader-willi syndrome: Challenges and solutions. Ther. Clin. Risk Manag..

[120] Tauber M., Diene G., Swaab D.F., Buijs R.M., Lucassen P.J., Salehi A., Kreier F. (2021). Chapter 26-Prader–Willi syndrome: Hormone therapies. Handbook of Clinical Neurology.

[121] Fountain M.D., Schaaf C.P. (2016). Prader-Willi Syndrome and Schaaf-Yang Syndrome: Neurodevelopmental Diseases Intersecting at the MAGEL2 Gene. Diseases.

[122] Schaaf C.P., Marbach F., Adam M.P., Mirzaa G.M., Pagon R.A., Wallace S.E., Bean L.J.H., Gripp K.W., Amemiya A. (1993). Schaaf-Yang Syndrome. GeneReviews ((R)).

[123] McCarthy J., Lupo P.J., Kovar E., Rech M., Bostwick B., Scott D., Kraft K., Roscioli T., Charrow J., Schrier Vergano S.A. (2018). Schaaf-Yang syndrome overview: Report of 78 individuals. Am. J. Med. Genet. A.

[124] Mejlachowicz D., Nolent F., Maluenda J., Ranjatoelina-Randrianaivo H., Giuliano F., Gut I., Sternberg D., Laquerriere A., Melki J. (2015). Truncating Mutations of MAGEL2, a Gene within the Prader-Willi Locus, Are Responsible for Severe Arthrogryposis. Am. J. Hum. Genet..

[125] Negishi Y., Kurosawa K., Takano K., Matsubara K., Nishiyama T., Saitoh S. (2022). A nationwide survey of Schaaf-Yang syndrome in Japan. J. Hum. Genet..

[126] Dykens E.M., Lee E., Roof E. (2011). Prader-Willi syndrome and autism spectrum disorders: An evolving story. J. Neurodev. Disord..

[127] Buiting K., Di Donato N., Beygo J., Bens S., von der Hagen M., Hackmann K., Horsthemke B. (2014). Clinical phenotypes of MAGEL2 mutations and deletions. Orphanet J. Rare Dis..

[128] Matarazzo V., Muscatelli F. (2013). Natural breaking of the maternal silence at the mouse and human imprinted Prader-Willi locus: A whisper with functional consequences. Rare Dis..

[129] Juriaans A.F., Kerkhof G.F., Garrelfs M., Trueba-Timmermans D., Hokken-Koelega A.C.S. (2023). Schaaf-Yang syndrome: Clinical phenotype and effects of 4 years of growth hormone treatment. Horm. Res. Paediatr..

[130] Hebach N.R., Caro P., Martin-Giacalone B.A., Lupo P.J., Marbach F., Choukair D., Schaaf C.P. (2021). A retrospective analysis of growth hormone therapy in children with Schaaf-Yang syndrome. Clin. Genet..

[131] McCarthy J.M., McCann-Crosby B.M., Rech M.E., Yin J., Chen C.A., Ali M.A., Nguyen H.N., Miller J.L., Schaaf C.P. (2018). Hormonal, metabolic and skeletal phenotype of Schaaf-Yang syndrome: A comparison to Prader-Willi syndrome. J. Med. Genet..

[132] Tauber M., Hoybye C. (2021). Endocrine disorders in Prader-Willi syndrome: A model to understand and treat hypothalamic dysfunction. Lancet Diabetes Endocrinol..

[133] Alves C., Franco R.R. (2020). Prader-Willi syndrome: Endocrine manifestations and management. Arch. Endocrinol. Metab..

[134] Hirsch H.J., Eldar-Geva T., Bennaroch F., Pollak Y., Gross-Tsur V. (2015). Sexual dichotomy of gonadal function in Prader-Willi syndrome from early infancy through the fourth decade. Hum. Reprod..

[135] Kojima M., Kangawa K. (2005). Ghrelin: Structure and function. Physiol. Rev..

[136] Pellikaan K., Ben Brahim Y., Rosenberg A.G.W., Davidse K., Poitou C., Coupaye M., Goldstone A.P., Hoybye C., Markovic T.P., Grugni G. (2021). Hypogonadism in Adult Males with Prader-Willi Syndrome-Clinical Recommendations Based on a Dutch Cohort Study, Review of the Literature and an International Expert Panel Discussion. J. Clin. Med..

[137] Turkkahraman D., Sirazi E.C., Aykal G. (2022). Serum alpha-melanocyte-stimulating hormone (a-MSH), brain-derived neurotrophic factor (BDNF), and agouti-related protein (AGRP) levels in children with Prader-Willi or Bardet-Biedl syndromes. J. Endocrinol. Investig..

[138] Correa-da-Silva F., Fliers E., Swaab D.F., Yi C.X. (2021). Hypothalamic neuropeptides and neurocircuitries in Prader Willi syndrome. J. Neuroendocr..

[139] Bochukova E.G., Lawler K., Croizier S., Keogh J.M., Patel N., Strohbehn G., Lo K.K., Humphrey J., Hokken-Koelega A., Damen L. (2018). A Transcriptomic Signature of the Hypothalamic Response to Fasting and BDNF Deficiency in Prader-Willi Syndrome. Cell Rep..

[140] Bittel D.C., Kibiryeva N., McNulty S.G., Driscoll D.J., Butler M.G., White R.A. (2007). Whole genome microarray analysis of gene expression in an imprinting center deletion mouse model of Prader-Willi syndrome. Am. J. Med. Genet. A.

[141] Stefan M., Ji H., Simmons R.A., Cummings D.E., Ahima R.S., Friedman M.I., Nicholls R.D. (2005). Hormonal and metabolic defects in a prader-willi syndrome mouse model with neonatal failure to thrive. Endocrinology.

[142] Gajewska J., Szamotulska K., Klemarczyk W., Chelchowska M., Strucinska M., Ambroszkiewicz J. (2023). Circulating Levels of Nesfatin-1 and Spexin in Children with Prader-Willi Syndrome during Growth Hormone Treatment and Dietary Intervention. Nutrients.

[143] Bueno M., Esteba-Castillo S., Novell R., Gimenez-Palop O., Coronas R., Gabau E., Corripio R., Baena N., Vinas-Jornet M., Guitart M. (2016). Lack of Postprandial Peak in Brain-Derived Neurotrophic Factor in Adults with Prader-Willi Syndrome. PLoS ONE.

[144] Eddiry S., Diene G., Molinas C., Salles J., Auriol F.C., Gennero I., Bieth E., Skryabin B.V., Rozhdestvensky T.S., Burnett L.C. (2021). SNORD116 and growth hormone therapy impact IGFBP7 in Prader-Willi syndrome. Genet. Med..

[145] Butler M.G., Bittel D.C., Talebizadeh Z. (2004). Plasma peptide YY and ghrelin levels in infants and children with Prader-Willi syndrome. J. Pediatr. Endocrinol. Metab..

[146] Haqq A.M., Muehlbauer M., Svetkey L.P., Newgard C.B., Purnell J.Q., Grambow S.C., Freemark M.S. (2007). Altered distribution of adiponectin isoforms in children with Prader-Willi syndrome (PWS): Association with insulin sensitivity and circulating satiety peptide hormones. Clin. Endocrinol..

[147] Butler M.G., Nelson T.A., Driscoll D.J., Manzardo A.M. (2015). Evaluation of Plasma Substance P and Beta-Endorphin Levels in Children with Prader-Willi Syndrome. J. Rare Disord..

[148] de Lind van Wijngaarden R.F., Otten B.J., Festen D.A., Joosten K.F., de Jong F.H., Sweep F.C., Hokken-Koelega A.C. (2008). High prevalence of central adrenal insufficiency in patients with Prader-Willi syndrome. J. Clin. Endocrinol. Metab..

[149] Nyunt O., Cotterill A.M., Archbold S.M., Wu J.Y., Leong G.M., Verge C.F., Crock P.A., Ambler G.R., Hofman P., Harris M. (2010). Normal cortisol response on low-dose synacthen (1 microg) test in children with Prader Willi syndrome. J. Clin. Endocrinol. Metab..

[150] Grugni G., Beccaria L., Corrias A., Crino A., Cappa M., De Medici C., Di Candia S., Gargantini L., Ragusa L., Salvatoni A. (2013). Central adrenal insufficiency in young adults with Prader-Willi syndrome. Clin. Endocrinol..

[151] Cataldi M., Arnaldi D., Tucci V., De Carli F., Patti G., Napoli F., Pace M., Maghnie M., Nobili L. (2021). Sleep disorders in Prader-Willi syndrome, evidence from animal models and humans. Sleep Med. Rev..

[152] Pace M., Falappa M., Freschi A., Balzani E., Berteotti C., Lo Martire V., Kaveh F., Hovig E., Zoccoli G., Amici R. (2020). Loss of Snord116 impacts lateral hypothalamus, sleep, and food-related behaviors. JCI Insight.

[153] Omokawa M., Ayabe T., Nagai T., Imanishi A., Omokawa A., Nishino S., Sagawa Y., Shimizu T., Kanbayashi T. (2016). Decline of CSF orexin (hypocretin) levels in Prader-Willi syndrome. Am. J. Med. Genet. A.

[154] Manzardo A.M., Johnson L., Miller J.L., Driscoll D.J., Butler M.G. (2016). Higher plasma orexin a levels in children with Prader-Willi syndrome compared with healthy unrelated sibling controls. Am. J. Med. Genet. A.

[155] Nevsimalova S., Vankova J., Stepanova I., Seemanova E., Mignot E., Nishino S. (2005). Hypocretin deficiency in Prader-Willi syndrome. Eur. J. Neurol..

[156] Kozlov S.V., Bogenpohl J.W., Howell M.P., Wevrick R., Panda S., Hogenesch J.B., Muglia L.J., Van Gelder R.N., Herzog E.D., Stewart C.L. (2007). The imprinted gene Magel2 regulates normal circadian output. Nat. Genet..

[157] Camerino C. (2023). Oxytocin’s Regulation of Thermogenesis May Be the Link to Prader-Willi Syndrome. Curr. Issues Mol. Biol..

[158] Miller J.L., Tamura R., Butler M.G., Kimonis V., Sulsona C., Gold J.A., Driscoll D.J. (2017). Oxytocin treatment in children with Prader-Willi syndrome: A double-blind, placebo-controlled, crossover study. Am. J. Med. Genet. A.

[159] Vaiani E., Herzovich V., Chaler E., Chertkoff L., Rivarola M.A., Torrado M., Belgorosky A. (2010). Thyroid axis dysfunction in patients with Prader-Willi syndrome during the first 2 years of life. Clin. Endocrinol..

[160] Sharkia M., Michaud S., Berthier M.T., Giguere Y., Stewart L., Deladoey J., Deal C., Van Vliet G., Chanoine J.P. (2013). Thyroid function from birth to adolescence in Prader-Willi syndrome. J. Pediatr..

[161] Lee H.J., Choe Y.H., Lee J.H., Sohn Y.B., Kim S.J., Park S.W., Son J.S., Kim S.W., Jin D.K. (2011). Delayed response of amylin levels after an oral glucose challenge in children with Prader-Willi syndrome. Yonsei Med. J..

[162] Butler M.G., Bittel D.C. (2007). Plasma obestatin and ghrelin levels in subjects with Prader-Willi syndrome. Am. J. Med. Genet. A.

[163] Park W.H., Oh Y.J., Kim G.Y., Kim S.E., Paik K.H., Han S.J., Kim A.H., Chu S.H., Kwon E.K., Kim S.W. (2007). Obestatin is not elevated or correlated with insulin in children with Prader-Willi syndrome. J. Clin. Endocrinol. Metab..

[164] DelParigi A., Tschop M., Heiman M.L., Salbe A.D., Vozarova B., Sell S.M., Bunt J.C., Tataranni P.A. (2002). High circulating ghrelin: A potential cause for hyperphagia and obesity in prader-willi syndrome. J. Clin. Endocrinol. Metab..

[165] Ding F., Li H.H., Zhang S., Solomon N.M., Camper S.A., Cohen P., Francke U. (2008). SnoRNA Snord116 (Pwcr1/MBII-85) deletion causes growth deficiency and hyperphagia in mice. PLoS ONE.

[166] Eldar-Geva T., Hirsch H.J., Rabinowitz R., Benarroch F., Rubinstein O., Gross-Tsur V. (2009). Primary ovarian dysfunction contributes to the hypogonadism in women with Prader-Willi Syndrome. Horm. Res..

[167] Bray G.A., Dahms W.T., Swerdloff R.S., Fiser R.H., Atkinson R.L., Carrel R.E. (1983). The Prader-Willi syndrome: A study of 40 patients and a review of the literature. Medicine.

[168] Butler M.G., Theodoro M., Skouse J.D. (2007). Thyroid function studies in Prader-Willi syndrome. Am. J. Med. Genet. A.

[169] Festen D.A., Visser T.J., Otten B.J., Wit J.M., Duivenvoorden H.J., Hokken-Koelega A.C. (2007). Thyroid hormone levels in children with Prader-Willi syndrome before and during growth hormone treatment. Clin. Endocrinol..

[170] Tauber M., Barbeau C., Jouret B., Pienkowski C., Malzac P., Moncla A., Rochiccioli P. (2000). Auxological and endocrine evolution of 28 children with Prader-Willi syndrome: Effect of GH therapy in 14 children. Horm. Res..

[171] McAlister K.L., Fisher K.L., Dumont-Driscoll M.C., Rubin D.A. (2018). The relationship between metabolic syndrome, cytokines and physical activity in obese youth with and without Prader-Willi syndrome. J. Pediatr. Endocrinol. Metab..

[172] Sohn Y.B., Kwak M.J., Kim S.J., Park S.W., Kim C.H., Kim M.Y., Kwon E.K., Paik K.H., Jin D.K. (2010). Correlation of adiponectin receptor expression with cytokines and insulin sensitivity in growth hormone (GH)-treated children with Prader-Willi syndrome and in non-GH-treated obese children. J. Clin. Endocrinol. Metab..

[173] Hoybye C., Bruun J.M., Richelsen B., Flyvbjerg A., Frystyk J. (2004). Serum adiponectin levels in adults with Prader-Willi syndrome are independent of anthropometrical parameters and do not change with GH treatment. Eur. J. Endocrinol..

[174] Pagano C., Marin O., Calcagno A., Schiappelli P., Pilon C., Milan G., Bertelli M., Fanin E., Andrighetto G., Federspil G. (2005). Increased serum resistin in adults with prader-willi syndrome is related to obesity and not to insulin resistance. J. Clin. Endocrinol. Metab..

[175] Haqq A.M., Muehlbauer M.J., Newgard C.B., Grambow S., Freemark M. (2011). The metabolic phenotype of Prader-Willi syndrome (PWS) in childhood: Heightened insulin sensitivity relative to body mass index. J. Clin. Endocrinol. Metab..

[176] Lindgren A.C., Marcus C., Skwirut C., Elimam A., Hagenas L., Schalling M., Anvret M., Lonnqvist F. (1997). Increased leptin messenger RNA and serum leptin levels in children with Prader-Willi syndrome and nonsyndromal obesity. Pediatr. Res..

[177] Butler M.G., Moore J., Morawiecki A., Nicolson M. (1998). Comparison of leptin protein levels in Prader-Willi syndrome and control individuals. Am. J. Med. Genet..

[178] Goldstone A.P., Brynes A.E., Thomas E.L., Bell J.D., Frost G., Holland A., Ghatei M.A., Bloom S.R. (2002). Resting metabolic rate, plasma leptin concentrations, leptin receptor expression, and adipose tissue measured by whole-body magnetic resonance imaging in women with Prader-Willi syndrome. Am. J. Clin. Nutr..

[179] Gu L., Ma Y., Gu M., Zhang Y., Yan S., Li N., Wang Y., Ding X., Yin J., Fan N. (2015). Spexin peptide is expressed in human endocrine and epithelial tissues and reduced after glucose load in type 2 diabetes. Peptides.

[180] Tauber M., Cutfield W. (2007). KIGS highlights: Growth hormone treatment in Prader-Willi Syndrome. Horm. Res..

[181] Noordam C., Hoybye C., Eiholzer U. (2021). Prader-Willi Syndrome and Hypogonadism: A Review Article. Int. J. Mol. Sci..

[182] Mercer R.E., Wevrick R. (2009). Loss of magel2, a candidate gene for features of Prader-Willi syndrome, impairs reproductive function in mice. PLoS ONE.

[183] Li K., Zheng X., Tang H., Zang Y.S., Zeng C., Liu X., Shen Y., Pang Y., Wang S., Xie F. (2021). E3 ligase MKRN3 is a tumor suppressor regulating PABPC1 ubiquitination in non-small cell lung cancer. J. Exp. Med..

[184] Miller J.L., Goldstone A.P., Couch J.A., Shuster J., He G., Driscoll D.J., Liu Y., Schmalfuss I.M. (2008). Pituitary abnormalities in Prader-Willi syndrome and early onset morbid obesity. Am. J. Med. Genet. A.

[185] Nillni E.A. (2010). Regulation of the hypothalamic thyrotropin releasing hormone (TRH) neuron by neuronal and peripheral inputs. Front. Neuroendocr..

[186] Tennese A.A., Wevrick R. (2011). Impaired hypothalamic regulation of endocrine function and delayed counterregulatory response to hypoglycemia in Magel2-null mice. Endocrinology.

[187] Scaroni C., Ceccato F., Rizzati S., Mantero F. (2008). Concomitant therapies (glucocorticoids and sex hormones) in adult patients with growth hormone deficiency. J. Endocrinol. Investig..

[188] Farholt S., Sode-Carlsen R., Christiansen J.S., Ostergaard J.R., Hoybye C. (2011). Normal cortisol response to high-dose synacthen and insulin tolerance test in children and adults with Prader-Willi syndrome. J. Clin. Endocrinol. Metab..

[189] Cataletto M., Angulo M., Hertz G., Whitman B. (2011). Prader-Willi syndrome: A primer for clinicians. Int. J. Pediatr. Endocrinol..

[190] Muller T.D., Nogueiras R., Andermann M.L., Andrews Z.B., Anker S.D., Argente J., Batterham R.L., Benoit S.C., Bowers C.Y., Broglio F. (2015). Ghrelin. Mol. Metab..

[191] Kweh F.A., Miller J.L., Sulsona C.R., Wasserfall C., Atkinson M., Shuster J.J., Goldstone A.P., Driscoll D.J. (2015). Hyperghrelinemia in Prader-Willi syndrome begins in early infancy long before the onset of hyperphagia. Am. J. Med. Genet. A.

[192] Baribeau D.A., Anagnostou E. (2015). Oxytocin and vasopressin: Linking pituitary neuropeptides and their receptors to social neurocircuits. Front. Neurosci..

[193] Bachner-Melman R., Ebstein R.P. (2014). The role of oxytocin and vasopressin in emotional and social behaviors. Handb. Clin. Neurol..

[194] Swaab D.F., Purba J.S., Hofman M.A. (1995). Alterations in the hypothalamic paraventricular nucleus and its oxytocin neurons (putative satiety cells) in Prader-Willi syndrome: A study of five cases. J. Clin. Endocrinol. Metab..

[195] Hoybye C., Barkeling B., Espelund U., Petersson M., Thoren M. (2003). Peptides associated with hyperphagia in adults with Prader-Willi syndrome before and during GH treatment. Growth Horm. IGF Res..

[196] Johnson L., Manzardo A.M., Miller J.L., Driscoll D.J., Butler M.G. (2016). Elevated plasma oxytocin levels in children with Prader-Willi syndrome compared with healthy unrelated siblings. Am. J. Med. Genet. A.

[197] Martin A., State M., Anderson G.M., Kaye W.M., Hanchett J.M., McConaha C.W., North W.G., Leckman J.F. (1998). Cerebrospinal fluid levels of oxytocin in Prader-Willi syndrome: A preliminary report. Biol. Psychiatry.

[198] Dombret C., Nguyen T., Schakman O., Michaud J.L., Hardin-Pouzet H., Bertrand M.J., De Backer O. (2012). Loss of Maged1 results in obesity, deficits of social interactions, impaired sexual behavior and severe alteration of mature oxytocin production in the hypothalamus. Hum. Mol. Genet..

[199] Ates T., Oncul M., Dilsiz P., Topcu I.C., Civas C.C., Alp M.I., Aklan I., Ates Oz E., Yavuz Y., Yilmaz B. (2019). Inactivation of Magel2 suppresses oxytocin neurons through synaptic excitation-inhibition imbalance. Neurobiol. Dis..

[200] Da Prato L.C., Zayan U., Abdallah D., Point V., Schaller F., Pallesi-Pocachard E., Montheil A., Canaan S., Gaiarsa J.L., Muscatelli F. (2022). Early life oxytocin treatment improves thermo-sensory reactivity and maternal behavior in neonates lacking the autism-associated gene Magel2. Neuropsychopharmacology.

[201] Bertoni A., Schaller F., Tyzio R., Gaillard S., Santini F., Xolin M., Diabira D., Vaidyanathan R., Matarazzo V., Medina I. (2021). Oxytocin administration in neonates shapes hippocampal circuitry and restores social behavior in a mouse model of autism. Mol. Psychiatry.

[202] Rice L.J., Agu J., Carter C.S., Harris J.C., Nazarloo H.P., Naanai H., Einfeld S.L. (2023). The relationship between endogenous oxytocin and vasopressin levels and the Prader-Willi syndrome behaviour phenotype. Front. Endocrinol..

[203] Lopez M., Seoane L.M., Garcia Mdel C., Dieguez C., Senaris R. (2002). Neuropeptide Y, but not agouti-related peptide or melanin-concentrating hormone, is a target peptide for orexin-A feeding actions in the rat hypothalamus. Neuroendocrinology.

[204] Hayashi M., Miyata R., Tanuma N. (2011). Decrease in acetylcholinergic neurons in the pedunculopontine tegmental nucleus in a patient with Prader-Willi syndrome. Neuropathology.

[205] Bischof J.M., Stewart C.L., Wevrick R. (2007). Inactivation of the mouse Magel2 gene results in growth abnormalities similar to Prader-Willi syndrome. Hum. Mol. Genet..

[206] Fountain M.D., Tao H., Chen C.A., Yin J., Schaaf C.P. (2017). Magel2 knockout mice manifest altered social phenotypes and a deficit in preference for social novelty. Genes Brain Behav..

[207] Yoon H., Enquist L.W., Dulac C. (2005). Olfactory inputs to hypothalamic neurons controlling reproduction and fertility. Cell.

[208] Grant B.D., Donaldson J.G. (2009). Pathways and mechanisms of endocytic recycling. Nat. Rev. Mol. Cell Biol..

[209] Seaman M.N. (2012). The retromer complex-endosomal protein recycling and beyond. J. Cell Sci..

[210] Borgonovo B., Ouwendijk J., Solimena M. (2006). Biogenesis of secretory granules. Curr. Opin. Cell Biol..

[211] Kogel T., Gerdes H.H. (2010). Maturation of secretory granules. Results Probl. Cell Differ..

[212] Wang J., Fedoseienko A., Chen B., Burstein E., Jia D., Billadeau D.D. (2018). Endosomal receptor trafficking: Retromer and beyond. Traffic.

[213] Seaman M.N., Gautreau A., Billadeau D.D. (2013). Retromer-mediated endosomal protein sorting: All WASHed up!. Trends Cell Biol..

[214] Myers S.E., Davis A., Whitman B.Y., Santiago J.V., Landt M. (2000). Leptin concentrations in Prader-Willi syndrome before and after growth hormone replacement. Clin. Endocrinol..

[215] Schmidt O., Teis D. (2012). The ESCRT machinery. Curr. Biol..

[216] Mercer R.E., Michaelson S.D., Chee M.J., Atallah T.A., Wevrick R., Colmers W.F. (2013). Magel2 is required for leptin-mediated depolarization of POMC neurons in the hypothalamic arcuate nucleus in mice. PLoS Genet..

[217] Hill J.W., Williams K.W., Ye C., Luo J., Balthasar N., Coppari R., Cowley M.A., Cantley L.C., Lowell B.B., Elmquist J.K. (2008). Acute effects of leptin require PI3K signaling in hypothalamic proopiomelanocortin neurons in mice. J. Clin. Investig..

[218] Osborne D.G., Phillips-Krawczak C.A., Billadeau D.D. (2015). Monitoring receptor trafficking following retromer and WASH deregulation. Methods Cell Biol..

[219] Stern C., Schwarz S., Moser G., Cvitic S., Jantscher-Krenn E., Gauster M., Hiden U. (2021). Placental Endocrine Activity: Adaptation and Disruption of Maternal Glucose Metabolism in Pregnancy and the Influence of Fetal Sex. Int. J. Mol. Sci..

